# Oleic Acid-Coated Zinc Ferrite Nanocubes: A Promising Nanocarrier for Neuroblastoma Therapy

**DOI:** 10.1021/acsomega.5c08155

**Published:** 2025-12-15

**Authors:** Çiğdem Elif Akgün, Fazilet Mısra Özdemir, İrem Abaka, Aydan Gülsu, Turan Demircan

**Affiliations:** † Research Laboratories Center, Muğla Sıtkı Koçman University, Menteşe, Muğla 48100, Turkey; ‡ Department of Physics, Muğla Sıtkı Koçman University, Menteşe, Muğla 48100, Turkey; § Department of Molecular Biology and Genetics, Muğla Sıtkı Koçman University, Menteşe, Muğla 48100, Turkey; ∥ Department of Medical Biology, Muğla Sıtkı Koçman University, Menteşe, Muğla 48100, Turkey

## Abstract

This study focuses on the synthesis of OA@ZnFe_2_O_4_ NPs and the investigation of their structural, magnetic, and biological characteristics as well as their pH-responsive drug release behavior for potential biomedical use. The particles with an average particle size of ∼23 nm exhibited a single-phase spinel structure and superparamagnetic behavior. DOX was successfully loaded onto the NPs with a remarkably high efficiency (∼99%). In vitro release studies demonstrated that the OA@ZnFe_2_O_4_ NPs exhibited a pH-dependent release behavior in phosphate-buffered saline and citrate buffers. The release rate was highest at pH 4.5, followed by pH 6.5 and then pH 7.4 in both buffer media. The OA@ZnFe_2_O_4_ NPs (0.5 mg/mL) in citrate buffer at pH 4.5 demonstrated a markedly higher release efficiency, reaching approximately 77% cumulative release over ∼5 days. Cell imaging demonstrated efficient uptake of the DOX@OA@ZnFe_2_O_4_ NPs in SH-SY5Y neuroblastoma cells. Biocompatibility assays showed that the OA@ZnFe_2_O_4_ NPs were nontoxic to normal 3T3 fibroblasts, while the DOX@OA@ZnFe_2_O_4_ NPs exhibited strong anticancer activity comparable to free DOX. These findings support the potential of the OA@ZnFe_2_O_4_ NPs as a pH-sensitive nanocarrier for targeted neuroblastoma therapy with reduced systemic side effects.

## Graphical abstract

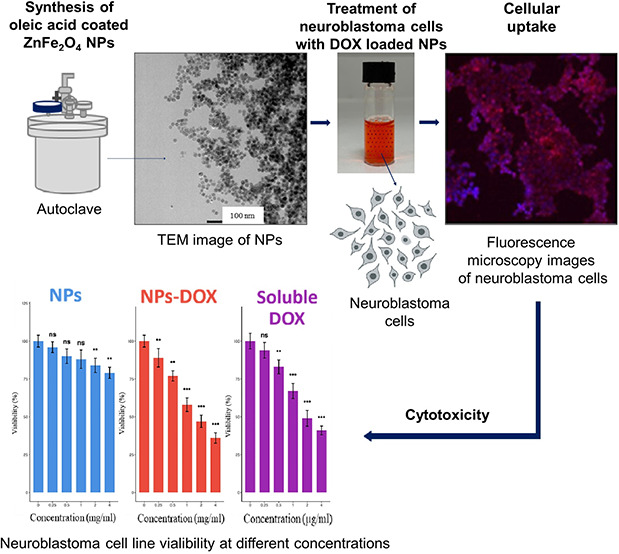

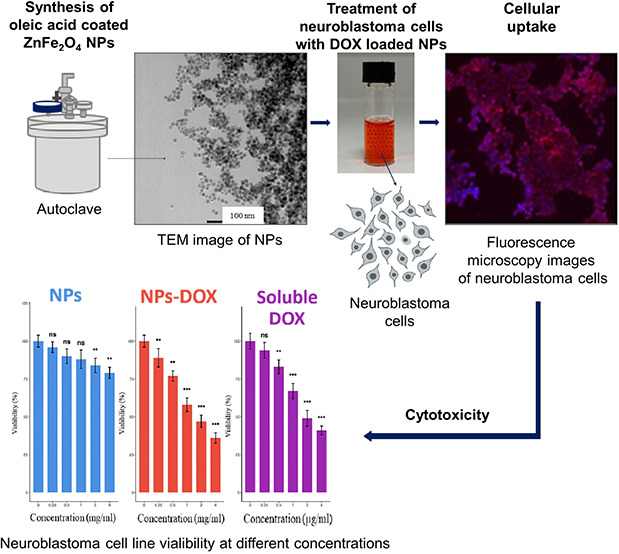

## 1 Introduction

Magnetic nanoparticles (MNPs) have become promising candidates for a range of biomedical applications, including drug delivery,^1^ magnetic resonance imaging,^2^ magnetic hyperthermia,^3^ and biosensing,^4^ due to unique physicochemical properties, such as small size, high surface area to volume ratio, and ease of surface functionalization.^5^ Among various types of MNPs, spinel ferrite NPs, particularly zinc ferrite (ZnFe_2_O_4_), have gained attention due to their biocompatibility, chemical stability, and tunable magnetic behaviors.^6–8^ Compared with classical inverse spinel Fe_3_O_4_, ZnFe_2_O_4_ exhibits a normal or mixed spinel structure. The partial substitution of Fe^2+^ and Fe^3+^ ions by Zn^2+^ ions in the spinel structure can cause lower magnetic coercivity, reduced remanence, and superparamagnetic behavior at room temperature. Due to cation distribution, the nanoparticle surface becomes enriched with Fe^3+^ and Zn^2+^ centers that act as Lewis acidic sites in association with hydroxyl groups (M–OH).^9^ Such surface sites exhibit strong affinity toward carboxylate- and amine-functionalized ligands, such as oleic acid (OA), chitosan, and peptides. The strong surface-ligand affinity supports the formation of dense and stable organic coatings and the efficient conjugation of therapeutic molecules. In addition to surface chemistry advantages, the biomedical applicability of ZnFe_2_O_4_ NPs is further supported by the favorable biological compatibility of Zn^2+^ ions with the human body.^10^ This makes the ZnFe_2_O_4_ NPs more favorable for in vivo use. However, like other ferrite MNPs (e.g., Fe_3_O_4_, CoFe_2_O_4_, and NiFe_2_O_4_), their surface still requires appropriate functionalization to prevent aggregation, enhance dispersibility, and optimize their interactions with therapeutic agents.^11–14^

To improve colloidal stability and biological performance, NPs are often functionalized with surface coatings with biocompatible agents such as carboxylic acids (e.g., OA^15^ and lauric acid^16^), tannic acid,^17^ polyethylene glycol,^18^ chitosan,^19^ etc. These surface coatings help minimize aggregation and prolonged circulation time and facilitate targeted delivery in biomedical applications. Among these, OA is a classical surfactant that anchors to the ferrite surface via carboxyl groups.^20^ OA forms a hydrophobic shell around the NPs, which enables strong interactions with hydrophobic drugs like doxorubicin (DOX) and facilitate efficient drug loading even in aqueous environments such as sodium borate buffer.^14^ DOX is a common chemotherapeutic agent used in the treatment of a variety of cancers, such as breast cancer,^21^ lung cancer,^22^ and neuroblastoma.^23^ Despite its effectiveness, its clinical use is often constrained by high systemic toxicity and a lack of tumor selectivity.^24^

NP-based delivery systems offer a promising strategy to overcome these limitations. These systems enable site-specific accumulation, controlled release, and prolonged circulation of the drug.^25–27^ pH-sensitive nanocarriers that release the drug preferentially in acidic environments, such as tumor tissues or intracellular compartments (e.g., endosomes and lysosomes), are highly desirable for targeted cancer therapy.^28^ To simulate physiological and pathological microenvironments during in vitro release studies, phosphate-buffered saline (PBS) and citrate buffer are commonly used as release media. Among them, PBS is composed mainly of monovalent ions and is commonly used to mimic physiological conditions due to its buffering capacity and ionic strength, similar to those of extracellular fluids. In contrast, citrate buffer provides a proton-rich, multivalent, and metal-chelating environment, which may substantially influence drug-NP interactions and the kinetics of drug release.^29,30^ Furthermore, NP concentration and surface charge play critical roles in modulating the desorption and diffusion of loaded drugs,^31,32^ yet the interplay among these parameters remains incompletely understood.

In this context, this study reports the fabrication of OA-coated ZnFe_2_O_4_ (OA@ZnFe_2_O_4_) NPs and explores their structural, magnetic, and biological features, including their controlled DOX release behavior under pH variations relevant to biomedical applications. The structural and magnetic properties of the NPs were analyzed to confirm their suitability for biomedical use. Drug loading efficiency, release profiles under varying pH (4.5, 6.5, and 7.4) and buffer conditions (PBS and citrate buffer), and in vitro cytotoxicity against noncancerous 3T3 fibroblast as a representative cell model and the SH-SY5Y neuroblastoma cancer cells were systematically investigated. This work aims to elucidate the potential of DOX-loaded OA@ZnFe_2_O_4_ (DOX@OA@ZnFe_2_O_4_) NPs for targeted cancer therapy with enhanced efficacy and reduced systemic toxicity.

## 2 Materials and Methods

### 2.1 Materials

Iron­(III) acetylacetonate (Merck, 97%), zinc acetate dihydrate (Merck, 99.5%), oleic acid (OA, Merck, technical grade, 90%), oleylamine (Merck, 70%), 1,2-hexadecanediol (Merck, 90%), dibenzyl ether (Merck, ≥98.0%), absolute ethanol (Merck, ≥99.5%), thiazolyl blue tetrazolium bromide (Thermo Scientific Chemicals, 98%), dimethyl sulfoxide (DMSO, PanReac AppliChem, Cell culture grade, 99.5%), penicillin/streptomycin (Pen/Strep, Capricorn Scientific, 100x), Dulbecco’s modified Eagle medium (DMEM, Gibco, with High Glucose, l-glutamine, Phenol Red, Sodium pyruvate and without *N*-(2-hydroxyethyl)­piperazine-*N*′-ethanesulfonic acid), trypsin–EDTA (Gibco, 0.25%, with phenol red), doxorubicin hydrochloride (DOX, Sigma-Aldrich, British Pharmacopoeia (BP) Reference Standard), fetal bovine serum (FBS, PAN-Biotech, South American origin, heat inactivated, 0.2 μm sterile filtered), and PBS tablets (PBS, BioShop) were purchased and used without purification.

### 2.2 Synthesis of the OA@ZnFe_2_O_4_ Nanoparticles

The OA@ZnFe_2_O_4_ NPs were synthesized using a convenient organic phase process as referenced by Sun et al.^33^ with slight modifications. The synthesis was performed under inert atmosphere (nitrogen) conditions using commercially available reagents. Zinc acetate dihydrate (1 mmol), iron (III) acetylacetonate (2 mmol), and 1,2-hexadecanediol (10 mmol) were weighed and mixed in the mixture of OA (6 mmol), oleylamine (6 mmol), and dibenzyl ether (6 mmol). The obtained mixture was enclosed in a stainless-steel autoclave with a volume of 100 mL. The air in the autoclave was excluded with nitrogen gas before the reaction. Then, the autoclave was sealed, heated to 200 °C and maintained at this temperature for 60 min. After that, the reaction temperature was raised to 260 °C and kept at this temperature for another 60 min. After the heat source was removed, the autoclave was cooled to room temperature naturally. The black colored powder of the OA@ZnFe_2_O_4_ NPs was collected by magnet and washed with ethanol several times to remove excess ligands. Finally, the washed product was dried at 40 °C overnight.

### 2.3 DOX Loading onto the OA@ZnFe_2_O_4_ Nanoparticles

DOX was loaded onto the OA@ZnFe_2_O_4_ NPs by using an incubation technique. Deionized (DI) water adjusted to pH 9 using 0.1 M NaOH solution was used as the loading medium. Alkaline conditions are known to enhance DOX sorption onto NP surfaces by promoting electrostatic interactions and improving drug-carrier affinity.^14,34^ For the loading process, 1 mL of a DOX solution at a concentration of 0.1 mg/mL was added to 10 mg of OA@ZnFe_2_O_4_ NPs (powder). The resulting suspension was first sonicated and then gently stirred using a rotator (DLAB MX-T6-S) at room temperature in the dark for 18 h. After mild stirring, the DOX@OA@ZnFe_2_O_4_ NPs were separated from the supernatant using an external magnet, washed with DI water 3 times, and subsequently dried in an incubator at 37 °C. The rotator was used instead of conventional magnetic stirring to ensure uniform dispersion and efficient drug loading. The rotator provides a gentle and continuous mixing that minimizes shear stress and prevents NP aggregation or sedimentation during the incubation period. This is particularly beneficial for OA-coated magnetic NPs, whose hydrophobic surfaces tend to aggregate or float in aqueous media such as DI water.

### 2.4 Characterization

#### 2.4.1 Structure, Morphology, and Magnetism

A variety of experimental techniques were used for structural, morphological, and magnetic characterizations of the OA@ZnFe_2_O_4_ NPs. The crystalline structure and phase purity of synthesized NPs were analyzed by x-ray powder diffraction (XRD) using a Rigaku SmartLab diffractometer. XRD patterns were collected on a zero-background quartz slide and under CuKα radiation in the range of 10–100°. The collected data was analyzed using the Rietveld refinement technique (FullProf program).^35,36^ Transmission electron microscope (TEM) images were captured using a FEI Talos F200S microscope to investigate the microstructure of the OA@ZnFe_2_O_4_ NPs. For the TEM sample preparation, the powder of the OA@ZnFe_2_O_4_ NPs was dispersed in ethanol and sonicated for 5 min. A drop from the very dilute suspension was then placed onto a carbon-coated copper grid and allowed to dry until evaporation of ethanol at ambient temperature. The number of above 100 NPs was determined from the TEM images to derive the particle size distribution. The average particle size and size distributions of the NPs were determined using ImageJ software (version 1.8.0).^37^ Fourier transform infrared spectroscopy (FTIR) data was recorded by using a Thermo Scientific Nicolet iS10 spectrometer to confirm the presence of the ZnFe_2_O_4_ NPs and OA coating on the NP surface. The FTIR spectrum was recorded in ATR mode in the wavenumber range 500–4000 cm^–1^. Thermogravimetric analysis (TGA) curves of NPs were obtained using a PerkinElmer STA 6000 thermal analyzer. The measurement was carried out along a temperature range of 30–800 °C with heating rate of 10 °C/min and under 20 mL/min flow of nitrogen atmosphere. A zeta sizer analyzer (Malvern Zetasizer NanoZSP) was used to measure the zeta potentials of the OA@ZnFe_2_O_4_ and DOX@OA@ZnFe_2_O_4_ NPs. For sample preparation, dilutions of NPs suspended in PBS (pH 4.5, to mimic the tumor environment) and in DMEM (supplemented with 10% FBS and %1 penicillin/streptomycin, to mimic physiological environment). Magnetic field-dependent magnetization curves were recorded using a Quantum Design PPMS DynaCool magnetometer between ±50 kOe at room temperature.

### 2.5 Drug Loading Efficiency and Releasing

DOX was used as a model anticancer agent to evaluate the drug loading capacity and subsequent drug release behavior of the OA@ZnFe_2_O_4_ NPs. To determine the drug loading efficiency (LE), the supernatant and washing solutions collected after magnetic separation were analyzed by using a UV–vis spectrophotometer (Thermo Scientific, Multiscan GO). A standard calibration was prepared by using a series of standard DOX solutions at known concentrations. The LE was determined using the following formula^38^

% LE=[(initialtotalDOXamount−freeDOXamountinsupernatant)/initialtotalDOXamount]×100

To evaluate the in vitro drug release behavior of the DOX@OA@ZnFe_2_O_4_ NPs under physiologically relevant conditions, two different buffer systems were used: PBS (at pH 4.5, 6.5, and 7.4) and citrate buffer (at pH 4.5, 6.5, and 7.4) at a nanoparticle concentration of 0.5 mg/mL. These pH values correspond to the endosomal/lysosomal (pH 4.5), tumor (pH 6.5), and physiological (pH 7.4) environments, respectively.^39^ All drug release studies were performed in a shaking water bath maintained at 37 °C. At predetermined time intervals, 0.5 mL of supernatants was withdrawn from each sample and immediately replaced with an equal volume of fresh prewarmed buffer to maintain a constant volume. Following magnetic separation of the NPs, the collected supernatants were analyzed by measuring the absorbance at 485 nm.

### 2.6 Drug Release Kinetics

To evaluate the release kinetics of DOX from the OA@ZnFe_2_O_4_ NPs, in vitro cumulative release data were analyzed over time. The percentage of drug released was plotted as a function of time, and the data were fitted to four different kinetic models: first-order, Higuchi, Korsmeyer-Peppas, and Weibull models to elucidate the release mechanism. These models were applied using the following equations^40–42^

$$First\;order,Q\left(t\right)=Q_{\infty }\left(1-\exp\left(-k_{1}t\right)\right)$$

$$Higuchi,Q\left(t\right)=k_{H}t^{1/2}$$

$$Korsmeyer-Peppas,Q\left(t\right)/Q_{\infty }=k_{KP}t^{n}$$

$$Weibull,Q\left(t\right)/Q_{\infty }=1-\exp\left(-at^{b}\right)$$

where *Q*(*t*) is the cumulative amount of DOX released at time *t*, *Q*(*t*)/*Q*_∞_ is the fractional release of DOX released at time *t*, *n* is the release exponent indicating the drug-release mechanism, *k*_1_, *k*_H_, and *k*_KP_ are the first-order, Higuchi, and Korsmeyer–Peppas release constants, respectively. In the Weibull model, *b* defines the profile of drug release and provides an inside look into the release mechanism. α reflects the intensity of the initial burst effect; higher α values correspond to faster initial release.^40^ The goodness of fit was evaluated by using the correlation coefficient *R* ^2^.

### 2.7 Cellular Internalization

To visualize nanoparticle internalization, the intrinsic autofluorescence of DOX was utilized as a fluorescent marker, enabling direct tracking of DOX-loaded nanoparticles within the cells without the need for additional labeling. SH-SY5Y cells were seeded at a density of 7 × 10^4^ cells/well in a 24-well plate and cultured in DMEM supplemented with 10% FBS and 1% penicillin/streptomycin at 37 °C under 5% CO_2_. After approximately 70–80% confluency was reached, the cells were treated with the OA@ZnFe_2_O_4_ and DOX@OA@ZnFe_2_O_4_ NPs for 24 h. Untreated cells served as controls. Following incubation, cells were washed twice with PBS and fixed with 10% formaldehyde for 30 min at room temperature. After washing, nuclei were stained using 300 nM DAPI solution (prepared by stepwise dilution from a 14.3 mM stock) and incubated for 15 min protected from light. Subsequently, the DAPI solution was removed, and cells were washed three times with PBS. Fluorescence images were acquired using a fluorescence microscope (Nikon ECLIPSE Ts2-FL). Overlay images were generated by merging DAPI and DOX channels to confirm localization of DOX.

### 2.8 Cytotoxicity Assay

The MTT assay was performed with slight modifications as described earlier.^43^ Both 3T3 fibroblast (noncancerous healthy cell line) and SH-SY5Y neuroblastoma cells were cultured in DMEM containing 10% FBS and 1% antibiotics (penicillin–streptomycin) under 37 °C and 5% CO_2_ conditions. Once the cells reached sufficient confluence, they were detached with trypsin–EDTA, centrifuged to remove the supernatant, and resuspended in a fresh medium. Cells were seeded into 96-well plates at a density of 1 × 10^4^ cells/well. The plates were incubated for 24 h to allow for cell attachment. Following incubation, three wells were designated as untreated controls for each plate, while the remaining wells were exposed to a concentration range of OA@ZnFe_2_O_4_ NPs (0.25–4 mg/mL) or DOX@OA@ZnFe_2_O_4_ NPs (equivalent NP concentrations of 0.25–4 mg/mL containing proportional DOX amounts). For comparison, free DOX was administered across a range of 0.25–4 μg/mL to evaluate dose-dependent cytotoxicity relative to the nanoparticle formulations. The treated cells were incubated for 24, 48, and 72 h. At the end of each period, the medium was aspirated, and 10 μL of 5 mg/mL MTT solution along with 100 μL of fresh medium was added to each well. The plates were incubated for 3–4 h. After incubation, the medium was removed, 100 μL of DMSO was added to each well to dissolve the formazan crystals, and the plates were gently shaken for 5 min in the dark using an orbital shaker. Absorbance was measured at 570 nm. The percentage of cytotoxicity was calculated using the following formula^44^

%cytotoxicity=[1−(meanopticaldensityoftreatedcells/meanopticaldensityofcontrolcells)]×100

### 2.9 Colony Formation Assay

The clonogenic potential of SH-SY5Y neuroblastoma cells following various treatments was assessed via colony formation assay (CFA) as described^45^ with minor adjustments. Cells were cultured under standard conditions as previously described.^45^ For the assay, 2000 cells per well were seeded into 6-well culture plates in triplicate and allowed to adhere overnight under standard culture conditions (37 °C, 5% CO_2_). The following day, cells were treated with one of the following: control medium (untreated), OA@ZnFe_2_O_4_ NPs (1 mg/mL), DOX@OA@ZnFe_2_O_4_ NPs (1 mg/mL), or soluble DOX (10 μg/mL). Throughout the 10 day incubation period, the culture medium was replaced every 4 days with fresh medium containing the respective treatments for the experimental groups or with treatment-free medium for the control group. After 10 days of incubation, colonies were fixed by using cold methanol for 15 min and stained with 0.5% crystal violet for 30 min at room temperature. Excess stains were removed by gentle rinsing with distilled water. Plates were air-dried and imaged under a stereomicroscope. The number of colonies in each well was quantified using the “ColonyCounter” plugin in ImageJ software (version 1.8.0),^37^ allowing for objective and standardized evaluation across conditions.

## 3 Results and Discussion

### 3.1 Structure and Morphology

The XRD pattern of the OA@ZnFe_2_O_4_ NPs is shown in Figure 1.

**Figure 1:**
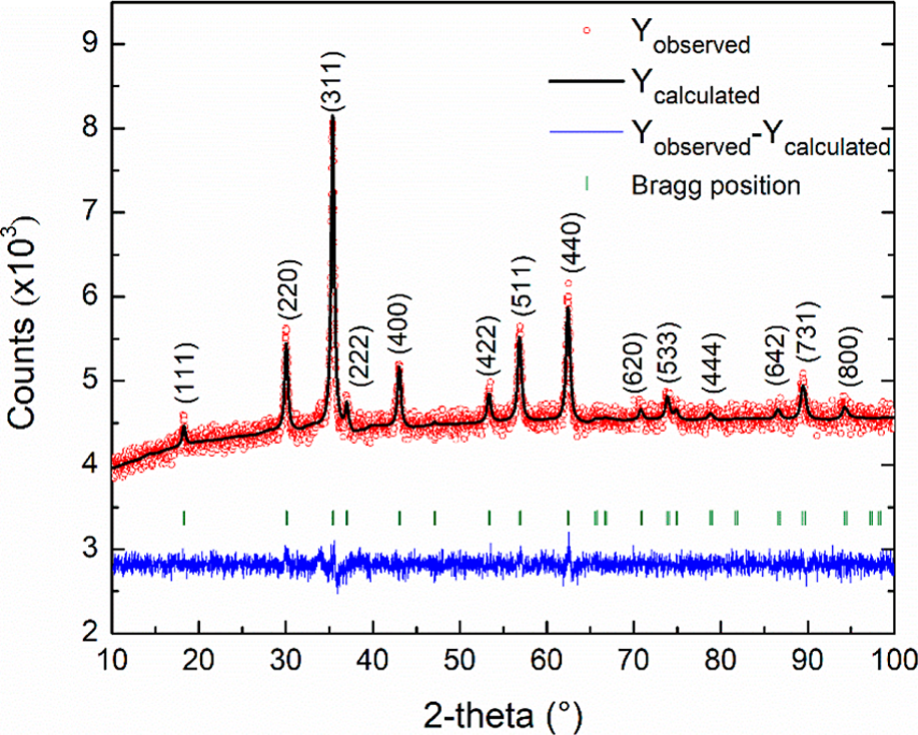
XRD pattern of the OA@ZnFe_2_O_4_ NPs, with the results of the Rietveld refinements (black lines). The Bragg markers identify the reflections (green) and the residuals to the refinement are presented below (blue lines).

The pattern exhibits typical diffraction peaks at 2θ values of 18.2° (111), 30.1° (220), 35.4° (311), 36.9° (222), 43.0° (400), 53.5° (422), 56.9° (511), 62.5° (440), 70.7° (620), 73.9° (533), and 78.8° (444), which are consistent with the reflections of a highly crystalline cubic spinel structure. These results align with the standard JCPDS card No. 82-1042,^46^ confirming the formation of single-phase ZnFe_2_O_4_ NPs with a space group of *Fd*3̅*m*. Rietveld refinement of the diffraction data yields lattice parameters of α = *b* = *c* = 8.4107(4) Å. The refinement also produced satisfactory agreement factors, with the value of 1.23, 1.54, 26.74, and 1.09 for *R*_p_, *R*_wp_, *R*_exp_, and χ^2^, respectively. Crystallite size estimation based on Scherrer broadening incorporated into the refinement indicates an average crystallite diameter of ∼15 nm. No secondary phases or impurities were observed in the XRD pattern.

Typical TEM images and the corresponding histogram plot of the sizes of the OA@ZnFe_2_O_4_ NPs are shown in Figure 2a–d.

**Figure 2:**
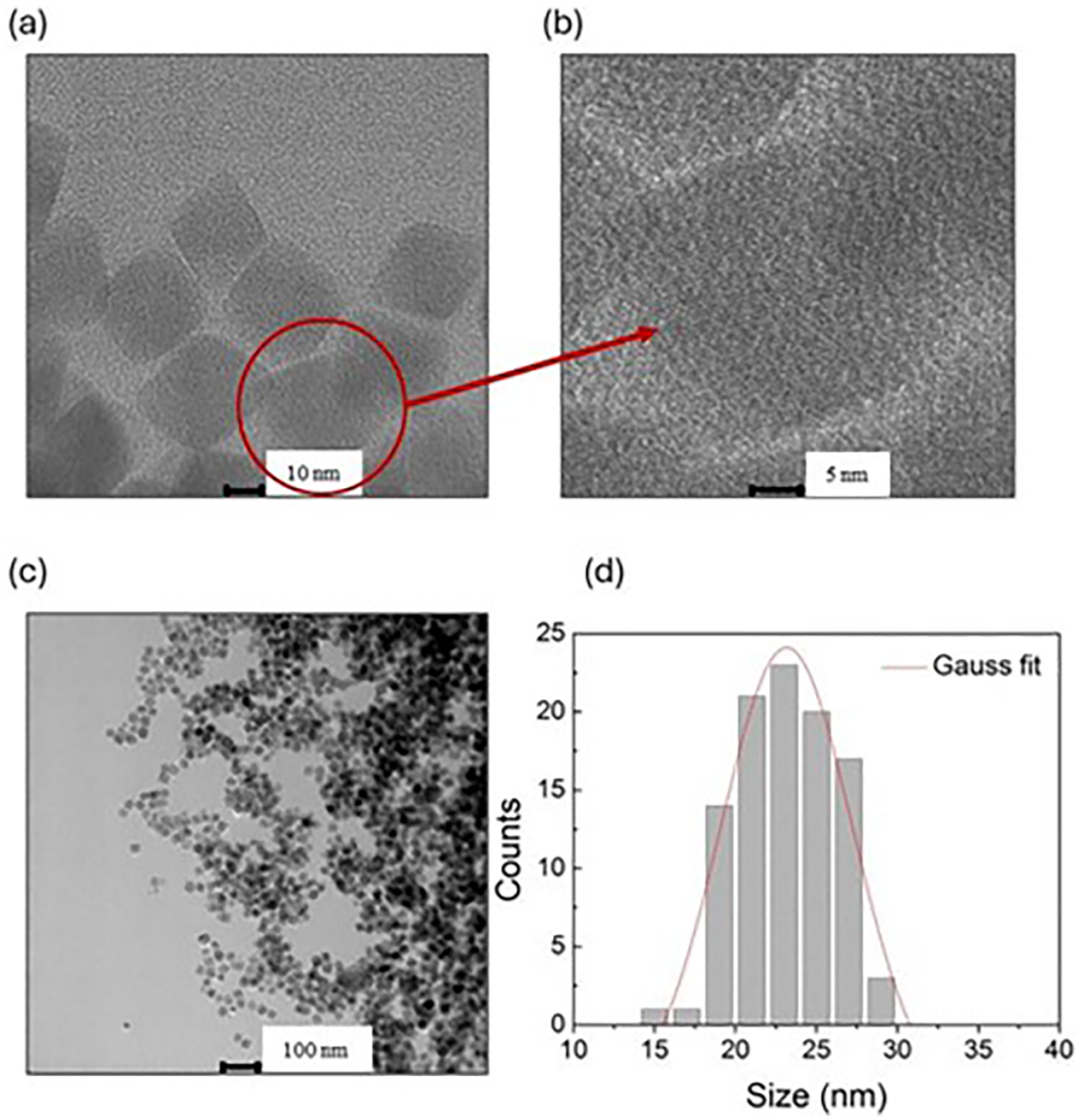
(a–c) TEM images and (d) histogram plot of the OA@ZnFe_2_O_4_ NPs.

As observed from Figure 2a–c, the OA@ZnFe_2_O_4_ NPs exhibit a uniform and well-defined cubic morphology with homogeneous dispersion. The histogram in Figure 2d was fitted with a Gaussian function, yielding an average particle diameter (⟨D⟩) with standard deviation (σ) as ⟨D⟩ = 23.15 ± 0.34 nm and σ = 4.06 for OA@ZnFe_2_O_4_ NPs. The surface morphology of the OA@ZnFe_2_O_4_ and DOX@OA@ZnFe_2_O_4_ NPs was also examined to examine the possible structural changes after DOX loading. The corresponding scanning electron microscopy (SEM) results confirmed the unchanged cubic morphology of the nanoparticles (Figure S1).

OA coating and DOX loading onto the OA@ZnFe_2_O_4_ NPs were confirmed by FTIR analysis. Figure 3a–c shows the FTIR spectra of the OA@ZnFe_2_O_4_ NPs, free DOX, and the DOX@OA@ZnFe_2_O_4_ NPs, respectively.

**Figure 3:**
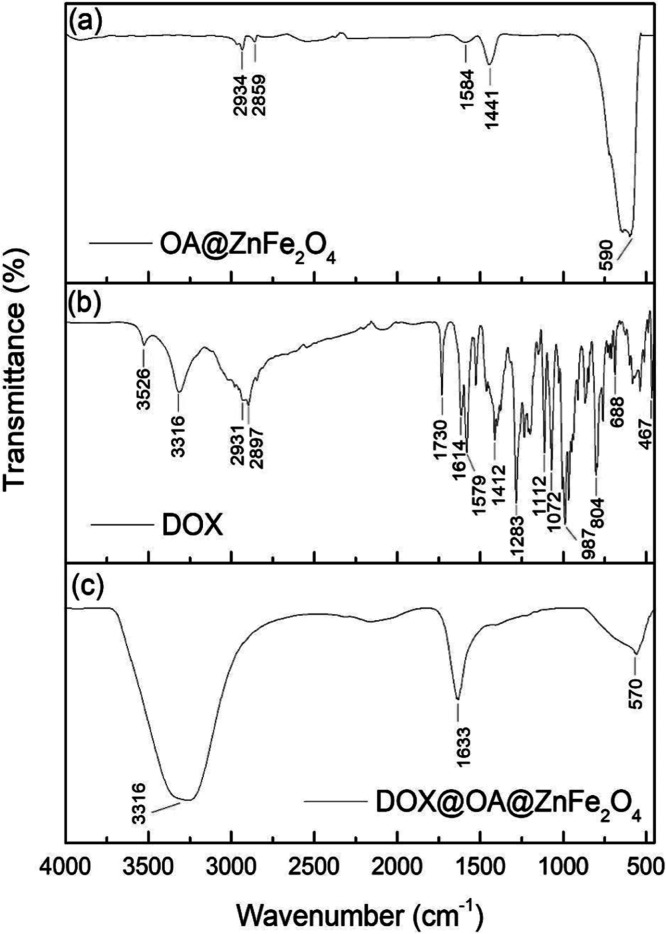
FTIR spectra of (a) OA@ZnFe_2_O_4_ NPs, (b) free DOX, and (c) DOX@OA@ZnFe_2_O_4_ NPs.

As seen from Figure 3a, the FTIR spectrum displays several characteristic vibrational bands that confirm the formation of the ferrite structure and the successful surface functionalization with OA. The peak observed at approximately 2934 cm^–1^ corresponds to the asymmetric stretching vibration of −CH_2_ groups, while the peak at 2859 cm^–1^ is attributed to the symmetric stretching of –CH_2_.^47^ These bands are indicative of the long hydrocarbon chain of OA, which suggests that the ZnFe_2_O_4_ NPs were effectively coated with OA. The distinct peaks at 1584 and 1441 cm^–1^ correspond to the asymmetric and symmetric stretching vibrations of the carboxylate (COO^–^) group, respectively.^48^ The separation between these two bands confirms the binding of oleate ions to the surface of the ZnFe_2_O_4_ NPs, likely through a bidentate or bridging coordination mode.^49^ Additionally, a strong absorption band centered at ∼590 cm^–1^ is assigned to the intrinsic metal–oxygen (Fe–O) vibration in the tetrahedral sites of the spinel ferrite lattice.^49^ The FTIR spectrum of the free drug DOX (Figure 3b) shows the stretching vibrations of the C=O and NH_2_ groups at 1614 and 3316 cm^–1^, respectively. The appearance of corresponding absorption bands at ∼1633 and ∼3316 cm^–1^ in the spectrum of the DOX@OA@ZnFe_2_O_4_ NPs (Figure 3c) confirms the successful loading of DOX.^50^

To determine the amount of adsorbed OA on the surface of the ZnFe_2_O_4_ NPs, TGA measurements were performed. Figure 4 shows the TGA curve of the OA@ZnFe_2_O_4_ NPs.

**Figure 4:**
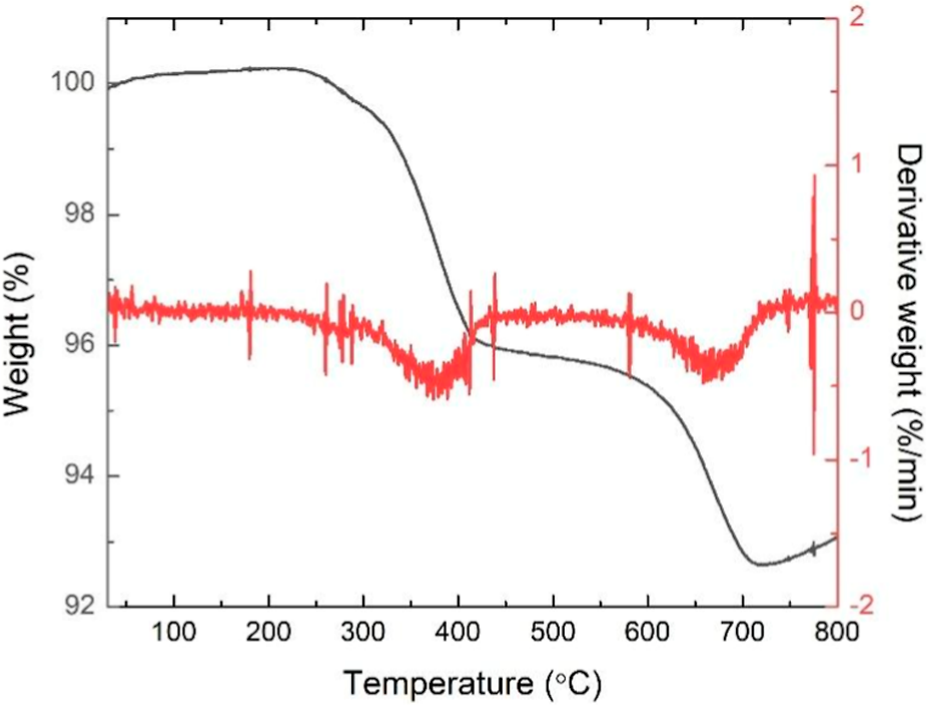
TGA curve of the OA@ZnFe_2_O_4_ NPs.

The TGA profile of the OA@ZnFe_2_O_4_ NPs revealed a total weight loss of ∼7.31%. The TGA curve revealed two separate mass loss steps: the first occurred in the 250–400 °C (low temperature) range and the second occurred in the 600–700 °C (high temperature) range. The mass loss step in low temperature range corresponds to weakly adsorbed (physically or secondarily bound) OA, while in high temperature range reflects the decomposition of tightly bound or chemically attached OA molecules to the surface of the ZnFe_2_O_4_ NPs.^51^ The TGA profile obtained for our OA@ZnFe_2_O_4_ NPs is consistent with previously reported studies, supporting the successful OA surface coating.^52^

### 3.2 Colloidal Stability of the Nanoparticles in Aqueous Solution

To evaluate the colloidal stability, the particle size and the surface charge of the OA@ZnFe_2_O_4_ and DOX@OA@ZnFe_2_O_4_ NPs were measured in PBS (pH 4.5) and DMEM. These analyses provided insight into their dispersion behavior and surface stability under acidic and physiological conditions. According to DLS measurements in PBS (pH 4.5), the *Z*-average hydrodynamic diameters were found to be 402.6 nm (PDI = 0.43) and 719.3 nm (PDI = 0.56) for the OA@ZnFe_2_O_4_ and DOX@OA@ZnFe_2_O_4_ NPs, respectively. The corresponding intensity-weighted distributions of the OA@ZnFe_2_O_4_ and DOX@OA@ZnFe_2_O_4_ NPs are presented in Figure S2a,b, respectively. However, the main intensity peaks were observed at ∼305 nm (for OA@ ZnFe_2_O_4_ NPs) and ∼440 nm (for DOX@OA@ZnFe_2_O_4_ NPs), which correspond to the dominant nanoparticle populations dispersed in PBS (pH 4.5). The higher *Z*-average of the DOX@OA@ZnFe_2_O_4_ NPs compared to the OA@ZnFe_2_O_4_ NPs can be attributed to the adsorption of DOX molecules onto the nanoparticle. Similar observations have been reported for DOX-loaded MNPs, where surface bound drug molecules lead to an increase in the measured hydrodynamic diameter.^14,53^

The hydrodynamic size distributions of the OA@ZnFe_2_O_4_ and DOX@OA@ZnFe_2_O_4_ NPs in DMEM are given in Figure S3a,b, respectively. The hydrodynamic size distributions of both OA@ZnFe_2_O_4_ and DOX@OA@ZnFe_2_O_4_ NPs exhibited a gradual decrease in the peak position. This indicates the improved colloidal stability during incubation in DMEM medium. For OA@ZnFe_2_O_4_ NPs, the initial distributions (1 h) were broad, with peaks around ∼2.5–3.5 × 10^3^ nm, corresponding to the presence of large aggregates resulting from hydrophobic interactions among OA layers and protein adsorption. Over time (from 1 to 6 h), the intensity and volume peaks progressively shifted toward smaller diameters (∼1.5–2.0 × 10^3^ nm), consistent with disaggregation and equilibration of the protein corona, which reduced hydrophobic bridging (a type of reversible noncovalent interactions) and enhanced dispersion stability. Such rearrangement is typical of soft corona formation, where weakly bound proteins replace the initial hard corona components over time. Compared to OA@ZnFe_2_O_4_ NPs, DOX@OA@ZnFe_2_O_4_ NPs exhibited smaller and narrower distributions at all time points, with peaks mainly located between 1.0 and 3.0 × 10^3^ nm. The reduced hydrodynamic size and narrower distribution can be attributed to the modification of the OA surface by DOX molecules, which increase hydrophilicity and introduce electrosteric stabilization. This modification suppresses protein-induced aggregation and promotes the formation of a more stable corona structure in DMEM medium. The variation in hydrodynamic size of the OA@ZnFe_2_O_4_ and DOX@OA@ZnFe_2_O_4_ NPs was monitored in DMEM over incubation periods ranging from 1 to 6 h to investigate the time-dependent formation of the protein corona in biological medium. These results are given in Figure 5.

**Figure 5:**
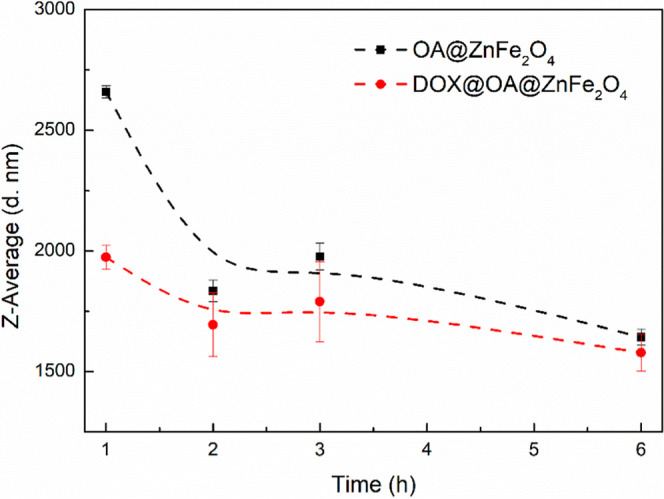
Variation in hydrodynamic size of the OA@ZnFe_2_O_4_ and DOX@OA@ZnFe_2_O_4_ NPs dispersed in DMEM over incubation periods ranging from 1 to 6 h, obtained from DLS measurements.

As seen from Figure 5, both nanoparticle systems show a time-dependent decrease in their hydrodynamic size. This behavior confirms the dynamic reorganization of the protein corona^54^ and the transition from a temporal aggregation-prone to a more stable dispersion. The smaller hydrodynamic sizes observed for the DOX@OA@ZnFe_2_O_4_ NPs compared to the OA@ZnFe_2_O_4_ NPs demonstrate that DOX loading improves the surface characteristics and colloidal stability of the nanoparticles in the DMEM medium. The adsorbed protein layer can electrostatically screen hydrophobic interactions of OA-coated NPs and prevent the large-scale agglomeration. It can be concluded that DOX loading may facilitate more uniform protein corona formation, which improves the colloidal dispersion of individual nanoparticles in DMEM medium. Furthermore, a notable reduction in Z-average values (from ∼2660 to 1818 nm for the OA@ZnFe_2_O_4_ NPs and from ∼1974 to 1688 nm for the DOX@OA@ZnFe_2_O_4_ NPs) was observed after 1 h of incubation. This time-dependent stabilization suggests that initial protein adsorption onto nanoparticles leads to the formation of a dynamic corona, which gradually rearranges into a more stable corona structure over time. After reaching an equilibration state, the hydrodynamic sizes remained nearly constant for both nanoparticle systems. This result confirms that a steady-state protein-coated dispersion was achieved. Such corona-mediated stabilization is advantageous in biological systems, as it enhances colloidal stability, reduces aggregation, and prolongs the circulation times of nanoparticles.

To further support the DLS results, the zeta potential of the OA@ZnFe_2_O_4_ and DOX@OA@ZnFe_2_O_4_ NPs was measured in PBS (pH 4.5) and DMEM, is given in Figure 6a,b, respectively.

**Figure 6:**
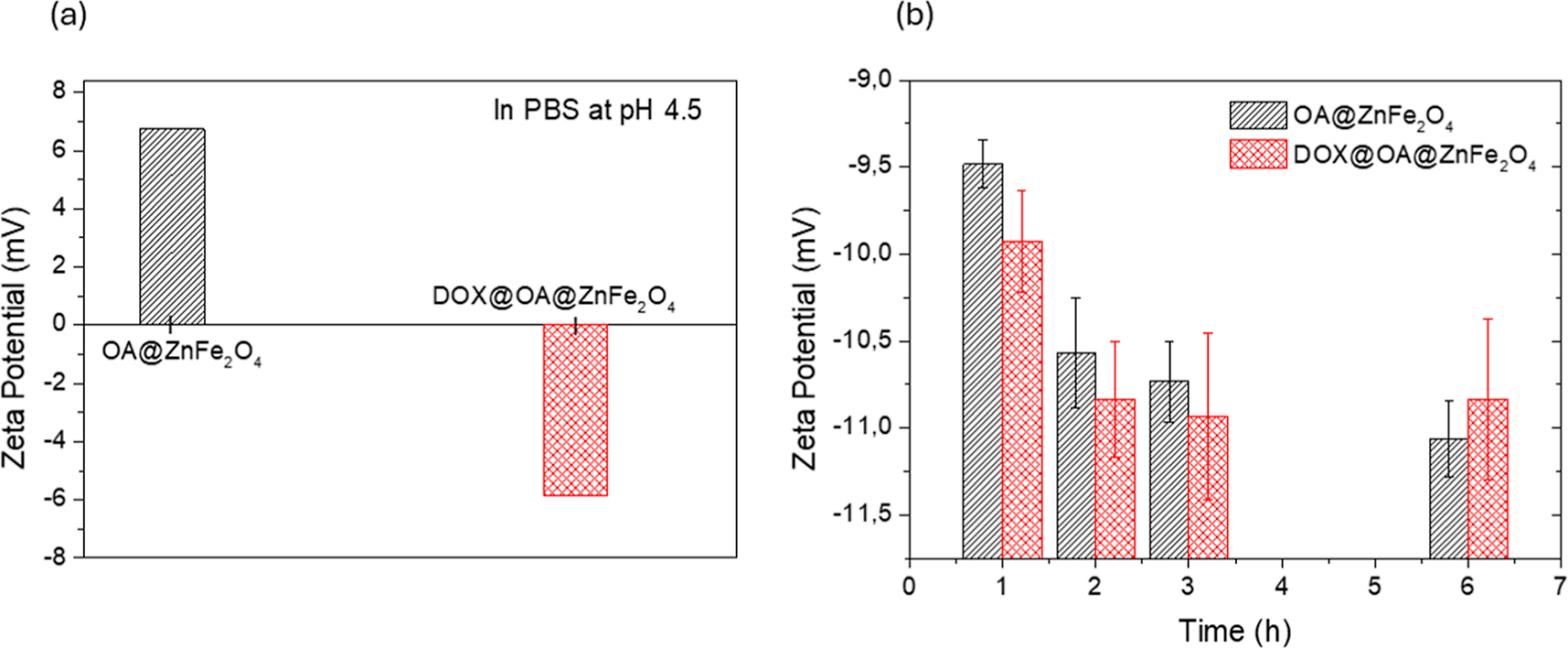
Zeta potentials of the OA@ZnFe_2_O_4_ and DOX@OA@ZnFe_2_O_4_ NPs in (a) PBS (pH 4.5) and (b) DMEM.

As seen from Figure 6a, the zeta potentials of the OA@ZnFe_2_O_4_ and DOX@OA@ZnFe_2_O_4_ NPs in PBS (pH 4.5) were found to be +6.74 and −5.84 mV, respectively. A notable shift seen in surface charge from +6.74 to −5.84 mV after DOX loading is likely due to the presence of electronegative functional groups from DOX molecules interacting with the NP surface. Although DOX is protonated and thus positively charged at pH 4.5 (p*K*_a_ ≈ 8.2), Nguyen et al. has reported negative zeta potential values (−27.4 mV) for DOX-functionalized NPs in acidic buffers.^55^ The reversal in surface charge can be attributed to the strong adsorption of DOX molecules onto the surface of the OA@ZnFe_2_O_4_ NPs. This may lead to the displacement or rearrangement of OA chains and contribute negatively charged functional groups. The pronounced shift supports that DOX is not loosely bound but instead forms a surface-associated layer.

As seen in Figure 6b, the zeta potential of the OA@ZnFe_2_O_4_ and DOX@OA@ZnFe_2_O_4_ NPs in DMEM exhibited comparable zeta potential values, which indicate that DOX loading did not affect the surface charge of the nanoparticles. In DMEM medium, both OA@ZnFe_2_O_4_ and DOX@OA@ZnFe_2_O_4_ NPs exhibited slightly negative surface charges of approximately −10.5 and −11.0 mV (on average), respectively. Additionally, no significant variation in surface charge was observed over time up to 6 h. Such stable values of zeta potential indicate that the nanoparticles maintained good stability in DMEM medium, where the adsorbed proteins formed a weak and dynamic corona layer without inducing significant charge reversal. The similarity of zeta potential values between the OA@ZnFe_2_O_4_ and DOX@OA@ZnFe_2_O_4_ NPs suggests that the DOX loading did not substantially alter the surface charge. This implies that the DOX molecules are partitioned into the hydrophobic OA shell around the nanoparticles via hydrophobic interactions (π–π and van der Waals interactions), so the aqueous interface seen by electrophoretic mobility is still dominated by OA (and any weakly bound proteins), not by DOX. Hence, zeta potential remains essentially unchanged. The unchanged zeta potential of the OA@ZnFe_2_O_4_ and DOX@OA@ZnFe_2_O_4_ NPs indicate that DOX loading does not appreciably alter the interfacial charge, consistent with DOX molecules partitioned into the hydrophobic OA shell rather than exposed at the aqueous interface, as reported for OA-coated iron oxide systems.^56,57^

Consequently, while the hydrodynamic size analysis revealed a time-dependent decrease of nanoparticles dispersed in DMEM, the nearly unchanged zeta potential values confirm that the nanoparticles maintained their colloidal stability without significant surface charge alteration or aggregation over time.

### 3.3 Magnetism

To understand the magnetization behavior of the OA@ZnFe_2_O_4_ NPs, the field (*H*) dependent magnetization (*M*) of the NPs was measured at 300 K under ±50 kOe applied fields. *M*–*H* curve at 300 K is shown in Figure 7.

**Figure 7:**
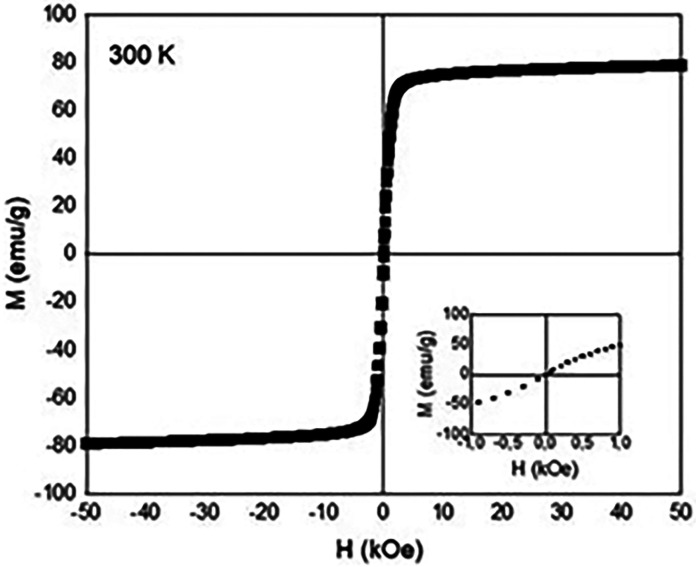
*M*–*H* curve of the OA@ZnFe_2_O_4_ NPs measured at 300 K.

The saturation magnetization (*M*_*s*_), remanant magnetization (*M*_*r*_) and coercivity (*H*_c_) values were recorded as ∼78 emu/g, ∼0.14 emu/g, and ∼0.024 kOe, respectively. It can be seen from these results that almost negligible remanence and coercivity existed, indicating the superparamagnetic behavior of the OA@ZnFe_2_O_4_ NPs at room temperature. The high saturation magnetization of the OA@ZnFe_2_O_4_ NPs ensures strong magnetic responsiveness, which is essential for applications such as magnetic targeting or magnetic hyperthermia.

### 3.4 Drug Loading and Releasing

The efficiency of drug loading onto the OA@ZnFe_2_O_4_ NPs and the subsequent release of the loaded drug represent critical factors in evaluating the overall performance of the delivery system. To evaluate both the LE and the release profile of DOX, standard calibration curves were prepared using a series of DOX solutions with known concentrations in PBS and citrate buffers (Figure S4a,b). The LE of DOX was found to be ∼99% for the OA@ZnFe_2_O_4_ NPs, significantly exceeding the values typically reported for ZnFe_2_O_4_-based systems. In previous studies, ZnFe_2_O_4_ nanocarriers have generally exhibited moderate drug loading capacities of ∼45–80%.^58–60^ DOX is a primarily hydrophobic chemotherapeutic agent that contains functional groups capable of forming hydrogen bonds and engaging in electrostatic interactions. OA is a highly hydrophobic long-chain fatty acid, which is known to interact strongly with the anthracycline moiety of DOX through hydrophobic interactions.^14^ Therefore, the remarkably high drug loading efficiency observed in this study may be attributed to multiple noncovalent binding mechanisms, including hydrogen bonding, electrostatic attraction, and π–π stacking interactions between DOX and the surface of the OA@ZnFe_2_O_4_ NPs.^61^

It is known that the release profile can be influenced by the physiological environment, such as pH.^28^ The release profile is also affected by different buffer environments, since different buffer solutions have different ionic structures and potential to form complexes with the drug.^62,63^ In vitro drug release profiles of the DOX@OA@ZnFe_2_O_4_ NPs with concentrations of 0.5 mg/mL were evaluated in two different buffers of PBS and citrate under the same conditions (pH 4.5, 6.5, and 7.4). The results are shown in Figure 8a,b, respectively.

**Figure 8:**
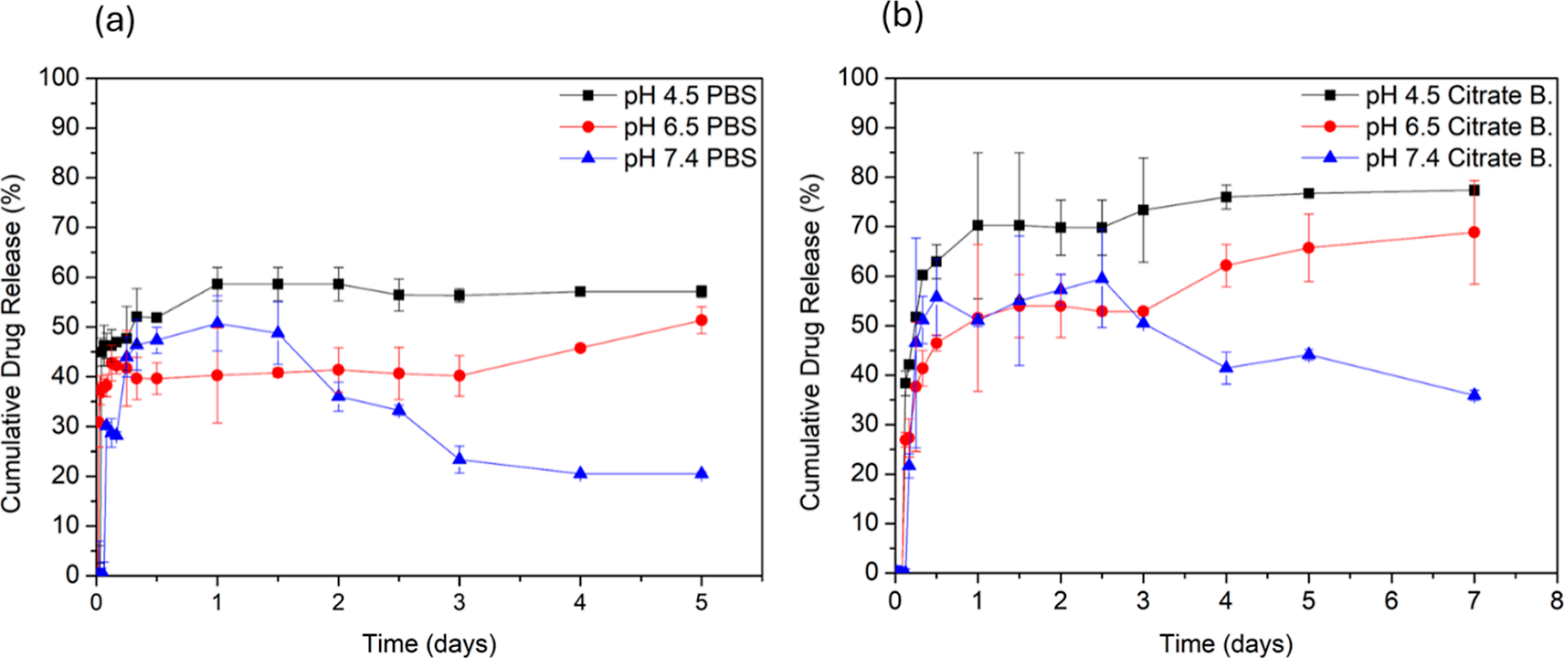
Cumulative drug release percentage versus time curves of DOX from the OA@ZnFe_2_O_4_ NPs at concentration of 0.5 mg/mL in (a) PBS and (b) citrate buffers at pH 4.5, 6.5, and 7.4.

In PBS-based release experiments, a clear pH-dependent release behavior was observed, as shown in Figure 8a. At pH 7.4, no detectable DOX release was observed within the first 120 min. Then, the release rate reached ∼40% in approximately 20 h. After that, the particle retained the drug.^64^ This is attributed to the particle’s lack of pH sensitivity at this pH. The coating material, OA, is nonionizable at pH 7.4 (p*K*_a_ ≈ 9.85), so release is low and slow.^65,66^ These experimental results demonstrate the long-term stability of NPs in the blood. Acidic media were used to simulate the intracellular conditions of cancer cells. At pH 6.5, ∼30% of the drug was released from the particles by 30 min, followed by sustained release, and the release rate reached ∼50% by day 5. As a result of OA protonation under these conditions, some hydrogen bonds and interactions may weaken at this pH. Consequently, release occurred largely by passive diffusion. Additionally, DOX has a moderate solubility at pH 6.5. Therefore, the release rate is slower. This allows for low-level passive release in environments such as a slightly acidic tumor microenvironment.

In controlled drug release studies, the release profile at pH 4.5 buffer conditions provides information about drug release from particles in an acidic microenvironment. The release studies observed at an acidic pH are important for targeting the particles to cancer cells.^67^ In this study, at pH 4.5 in PBS, ∼45% of the drug was released in the first 60 min. This was followed by a controlled release over 4 days. Approximately ∼60% of the drug was released by the end of day 5. This was attributed to the increased protonation and hydrophobicity of the OA-coated nanoparticles, allowing the diffusion of the drug from the particles.

In addition to observing the release profile in PBS, release studies were conducted in citrate buffer to observe the effect of the buffer solution on the drug release rate. The release profile of the DOX@OA@ZnFe_2_O_4_ NPs in citrate buffer at different pH values (pH 4.5, 6.5, and 7.4) is shown in Figure 8 (b). As it is seen in the Figure 8 (b), ∼20% of the drug was released within the first 250 min in the pH 7.4 citrate buffer. Controlled release started at approximately 500 min and continued until day 3. Then, as in PBS, the drug started to accumulate back into the drug carrier. Approximately ∼35% of the drug was released on day 7. As noted in the PBS medium, the release rate is slow under these conditions due to low pH sensitivity. The coating material, OA, is nonionizable at pH 7.4 (p*K*_a_ ≈ 9.85), resulting in low and slow release.^65,66^ All of these experimental results demonstrate the stability of the OA@ZnFe_2_O_4_ NPs in blood for extended periods.

Experimental studies continued in a citrate buffer at pH 6.5. Controlled and sustained release was observed starting at 500 min ∼70% of the drug released by the end of day 7. Similar to the behavior observed in PBS, the OA@ZnFe_2_O_4_ NPs are partially protonated under these conditions. At this pH, the electrostatic interactions between DOX and the nanoparticle surface are weakened. Considering the moderate solubility of DOX in the buffer solution at pH 6.5, the observed release profile can be attributed to a diffusion-controlled drug release.

In citrate buffer medium at pH 4.5, controlled release started after the 500^th^ minute, and it was observed that ∼ 80% of the drug was released at the end of the 7^th^ day.

In summary, similar procedures were followed to determine the release pattern of DOX from the optimized DOX@OA@ZnFe_2_O_4_ NP formulation in different buffers (having different pH). pH-dependent release behavior was observed in citrate buffer, just like in PBS. The release rate of DOX from the OA@ZnFe_2_O_4_ NPs increased with decreasing pH. When comparing DOX release from the OA@ZnFe_2_O_4_ NPs in PBS and citrate buffer, release is lower in PBS due to the hydrophobicity of OA and the low solubility of DOX in PBS.

### 3.5 Drug Release Kinetics

The DOX release curve fittings in PBS and citrate buffers (at pH 4.5, 6.5, and 7.4) are shown in Figure 9a,b, respectively. The corresponding kinetic parameters are summarized in Table 1.

**Figure 9:**
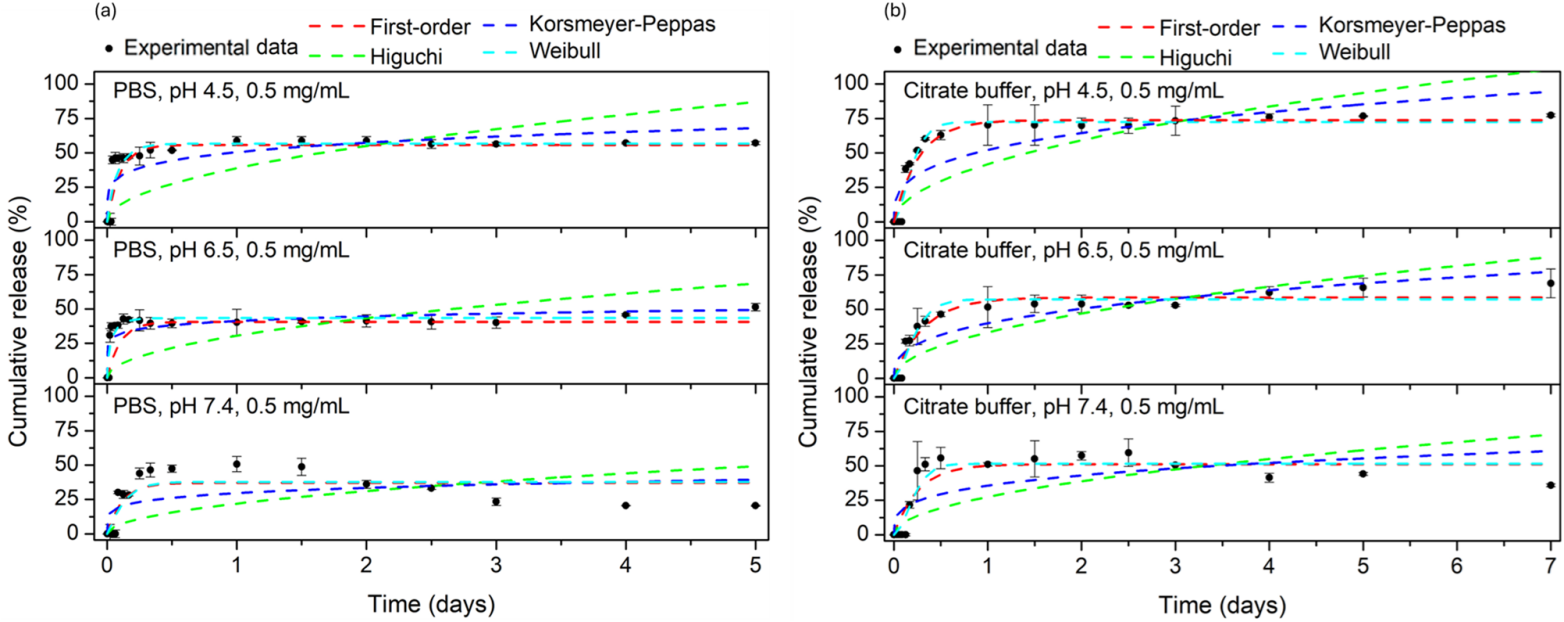
Kinetics study of DOX release from the OA@ZnFe_2_O_4_ NPs (0.5 mg/mL) in (a) PBS and (b) citrate buffers at pH 4.5, 6.5, and 7.4 by applying first-order, Higuchi, Korsmeyer–Peppas and Weibull models, respectively.

**Table 1:** Kinetic Fitting Parameters of DOX Release from the OA@ZnFe_2_O_4_ NPs (0.5 mg/mL) in PBS and Citrate Buffers at pH 4.5, 6.5, and 7.4 Based on First-Order, Higuchi, Korsmeyer–Peppas, and Weibull Models

condition	first-order *k*_1_	first-order *R* ^2^	Higuchi k_H_	Higuchi *R* ^2^	Korsmeyer–Peppas *k*_KP_, *n*	Korsmeyer–Peppas *R* ^2^	Weibull α, *b*	Weibull *R* ^2^
PBS pH 4.5	10.000 ± 2.465	0.820	38.888 ± 5.129	0.035	0.505 ± 0.038	0.624	10.000 ± 8.125	0.835
					0.185 ± 0.045		0.921 ± 0.276	
PBS pH 6.5	10.000 ± 4.256	0.324	30.644 ± 4.725	–0.963	0.413 ± 0.027	0.570	10.000 ± 8.921	0.774
					0.111 ± 0.033		0.680 ± 0.245	
PBS pH 7.4	8.567 ± 3.420	0.690	22.000 ± 4.405	–0.070	0.296 ± 0.042	0.319	10.000 ± 13.905	0.689
					0.177 ± 0.083		1.146 ± 0.573	
Citrate buffer pH 4.5	3.788 ± 0.567	0.944	41.848 ± 3.746	0.657	0.522 ± 0.042	0.777	10.000 ± 4.681	0.964
					0.305 ± 0.053		1.540 ± 0.260	
Citrate buffer pH 6.5	3.101 ± 0.487	0.940	33.257 ± 2.410	0.773	0.399 ± 0.287	0.843	7.370 ± 3.714	0.948
					0.340 ± 0.049		1.478 ± 0.299	
Citrate buffer pH 7.4	3.852 ± 1.040	0.845	27.450 ± 3.683	0.417	0.357 ± 0.043	0.566	10.000 ± 8.505	0.902
					0.274 ± 0.078		1.659 ± 0.510	

As seen from Table 1, the Weibull model provided the best correlation, with *R* ^2^ values of 0.835 and 0.964 for PBS and citrate buffers at pH 4.5, respectively. This was followed by the first-order model (with *R* ^2^ = 0.820 for PBS and *R* ^2^ = 0.944 for citrate buffer at pH 4.5), which also showed good agreements with the release profile of the OA@ZnFe_2_O_4_ NPs. In the Weibull model, the shape parameter *b* provides mechanistic information about the release process. Values of *b* < 0.75 are generally associated with Fickian diffusion-controlled release. In Fickian diffusion, an increase in the *b* value suggests that the medium becomes less disordered. The *b* values in the range of 0.75–1.00 reflect predominantly diffusive but slightly anomalous transport, while *b* > 1 indicates complex, non-Fickian release behavior governed by matrix relaxation processes and possible coating layer destabilization or surface erosion.^68–70^ In our study, the *b* values in PBS are in the range of ∼0.68–1.15 indicate mainly the Fickian diffusion-driven release profile at pH 6.5 and minor relaxation-related contributions at pH 4.5 and 7.4. In citrate buffer, the negligible variation in the *b* values among pH 4.5, 6.5, and 7.4 suggests that the release behavior was independent of pH. The higher *b* value in citrate buffer (∼1.54) compared to PBS (∼0.92) suggests a mechanistic shift toward non-Fickian anomalous release behavior of the OA@ZnFe_2_O_4_ NPs. This is likely from partial destabilization or relaxation- or erosion-controlled DOX release driven by citrate-ferrite complexation at the nanoparticle surface.

### 3.6 Cellular Uptake of the Nanoparticles and Cytotoxicity Assays

Following the characterization steps, the internalization capacity of NPs was investigated on SH-SY5Y cells (Figure 10).

**Figure 10:**
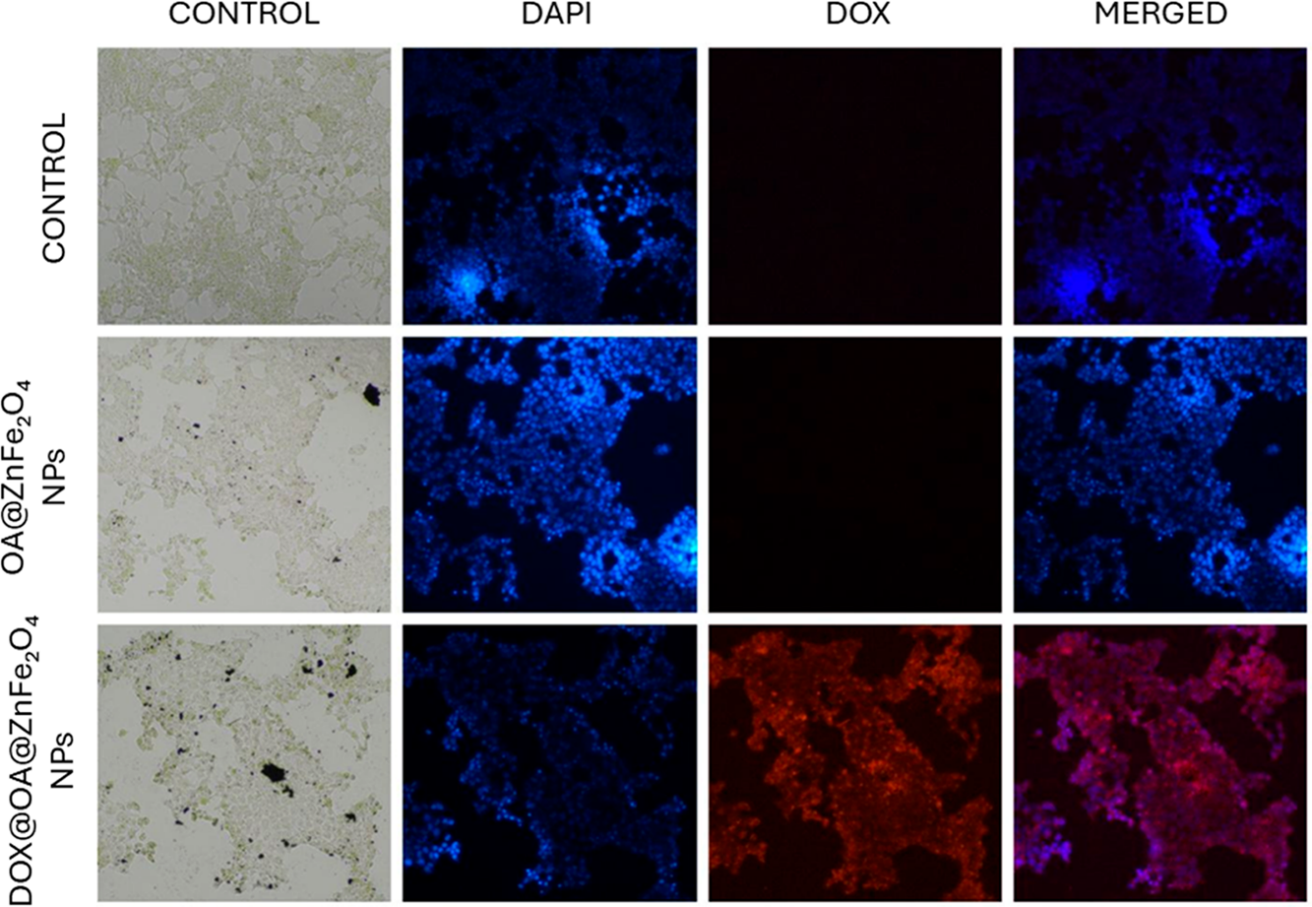
Fluorescence microscopy images of SH-SY5Y cells after 24 h incubation with control medium, OA@ZnFe_2_O_4_ NPs, and DOX@OA@ZnFe_2_O_4_ NPs (10× magnification). Nuclei were stained with DAPI (blue), while DOX fluorescence appears in red.

Cells treated with the DOX@OA@ZnFe_2_O_4_ NPs exhibit strong red fluorescence colocalized with DAPI-stained nuclei, indicating intracellular uptake of nanoparticles, whereas neither the control groups nor the OA@ZnFe_2_O_4_-only group exhibited detectable red emission (Figure 10). Importantly, no fluorescence was observed in cell-free regions of the substrate, suggesting that DOX molecules were not released extracellularly or adsorbed on the surface. This finding implies that the nanoparticles were first internalized by the cells and most probably followed by intracellular DOX release and nuclear accumulation.^53,71^ These results support that the comparable cytotoxicity of the DOX@OA@ZnFe_2_O_4_ NPs and free DOX arises from the efficient cellular uptake of the OA@ZnFe_2_O_4_ NPs and subsequent intracellular drug release rather than premature extracellular diffusion.

The cytocompatibility and selective cytotoxicity of the synthesized nanoparticles were evaluated using both a noncancerous cell line (3T3 fibroblasts) and a neuroblastoma cancer cell line (SH-SY5Y) across a range of concentrations (0.25–4 mg/mL; Figure 11).

**Figure 11:**
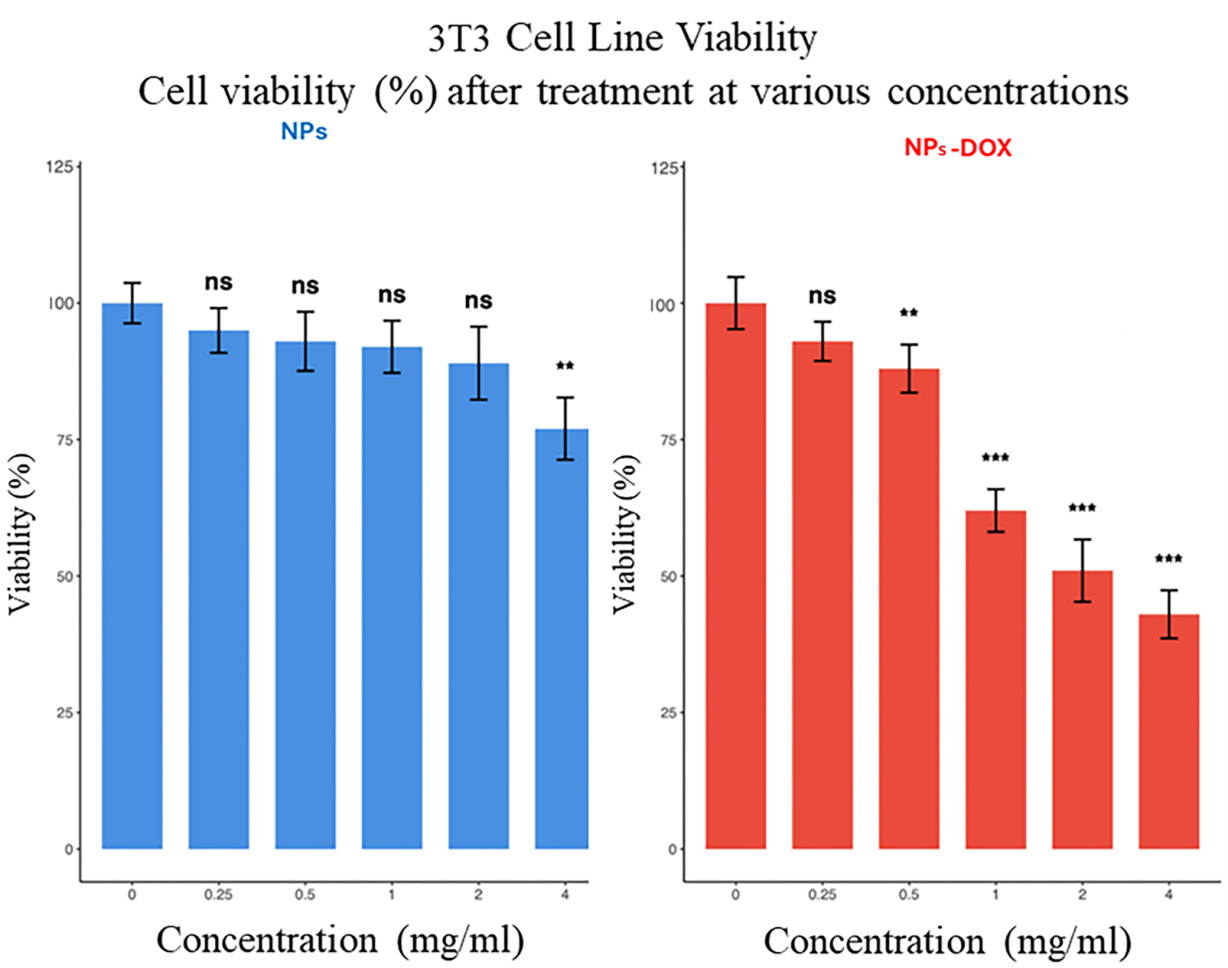
Cell viability of 3T3 fibroblasts after exposure to NPs (OA@ZnFe_2_O_4_) and NPs-DOX (DOX@OA@ZnFe_2_O_4_) at increasing concentrations (0.25–4 mg/mL). Data are presented as mean ± SD. For statistical comparisons with control (untreated group), *t*-test was conducted. ns: nonsignificant; **p < 0.01 and ***p < 0.001.

In 3T3 cells, treatment with bare OA@ZnFe_2_O_4_ NPs did not cause a significant reduction in viability at any tested concentration, maintaining cell viability above 89% even at higher doses (Figure 11). However, at the highest concentration (4 mg/mL), a mild reduction in viability was observed, with 77% cell survival (*p* < 0.01), indicating a slight dose-dependent effect. This finding indicates that the nanocarrier itself exhibits high biocompatibility with normal cells. On the other hand, exposure to DOX-loaded nanoparticles (DOX@OA@ZnFe_2_O_4_) resulted in a moderate, dose-dependent decrease in cell viability, reaching statistical significance at concentrations ≥0.5 mg/mL. The observed reduction in 3T3 cell viability is most likely attributed to the DOX content within the nanoparticles rather than the OA@ZnFe_2_O_4_ carrier itself, indicating that the mild toxicity arises from drug-related effects rather than intrinsic nanoparticle cytotoxicity (Figure 11).

In contrast, SH-SY5Y neuroblastoma cells displayed pronounced dose-dependent sensitivity to the DOX@OA@ZnFe_2_O_4_ NPs treatment (Figure 12).

**Figure 12:**
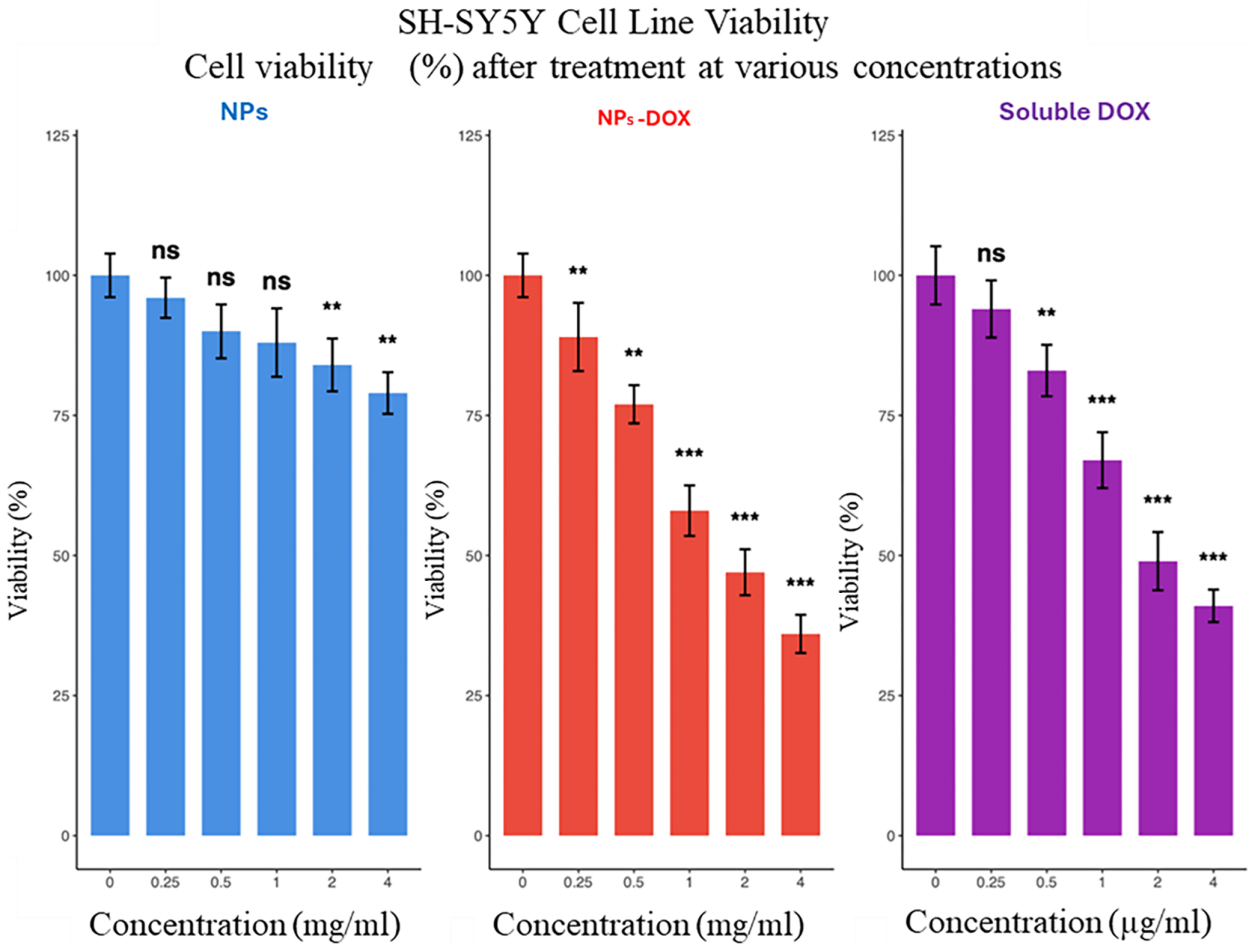
Cytotoxic effects of NPs (OA@ZnFe_2_O_4_), NPs-DOX (DOX@OA@ZnFe_2_O_4_), and free DOX on SH-SY5Y neuroblastoma cells. Cells were treated with increasing concentrations (0.25–4 mg/mL) of each formulation or soluble DOX (0.25–4 μg/mL) for 24 h, followed by MTT assay for viability determination. Data are presented as mean ± SD. Statistical analysis was performed using *t*-test and comparisons with control are indicated by asterisks. ns: nonsignificant; **p < 0.01 and ***p < 0.001.

Both free DOX and the DOX-loaded nanoparticles significantly decreased cell viability, particularly at concentrations above 0.5 mg/mL, where the viability dropped below 40%. Interestingly, the OA@ZnFe_2_O_4_ NPs alone caused only a minor but significant reduction in SH-SY5Y viability (for 2 and 4 mg/mL), suggesting that the core nanomaterial is not inherently toxic to cancer cells but serves as an effective delivery platform. The comparable cytotoxic profiles of the DOX@OA@ZnFe_2_O_4_ NPs and free DOX suggest efficient intracellular release of the drug following nanoparticle uptake, consistent with the internalization and nuclear colocalization data observed previously (Figure 10). Taken together, these results highlight the dual advantage of the OA@ZnFe_2_O_4_ nanocarrier system, which combines excellent biocompatibility toward normal cells with high therapeutic efficacy against cancer cells through targeted drug internalization and controlled intracellular DOX release.

Following the MTT assay, phase-contrast microscopy was performed to visualize morphological alterations induced by the treatments over 24, 48, and 72 h (Figure 13).

**Figure 13:**
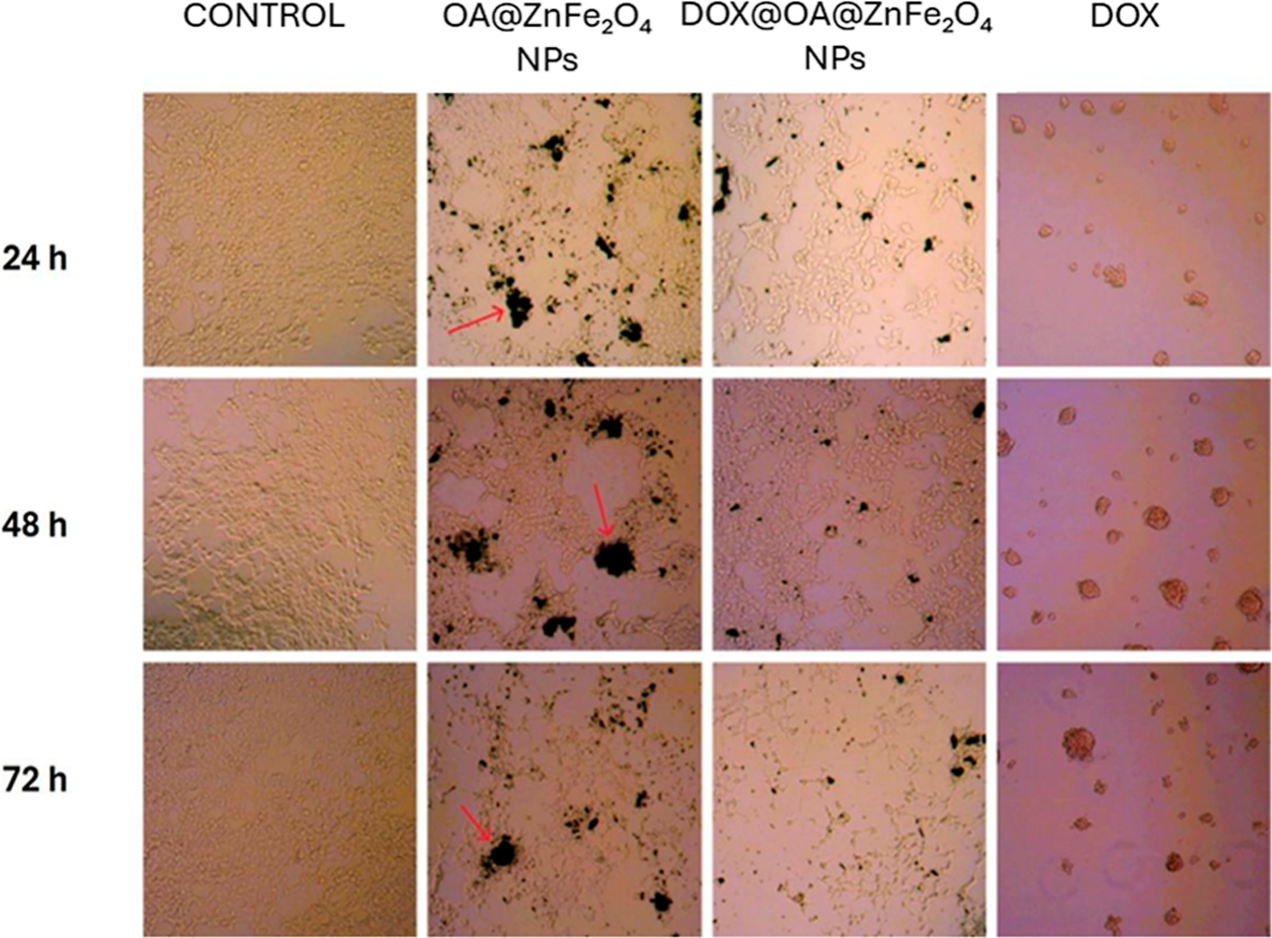
Microscopic images (10×) of SH-SY5Y cells after 24, 48, and 72 h of incubation with DOX, OA@ZnFe_2_O_4_ NPs, and DOX@OA@ZnFe_2_O_4_ NPs.

Untreated control cells maintained a confluent monolayer with a characteristic polygonal morphology and intact intercellular junctions throughout the observation period, indicating healthy growth conditions. In contrast, cells treated with the OA@ZnFe_2_O_4_ NPs exhibited mild morphological alterations, including scattered regions of cell detachment and occasional dark aggregates (marked by red arrows), likely corresponding to internalized or surface-bound nanoparticles. The DOX@OA@ZnFe_2_O_4_ NPs-treated cells displayed more pronounced cytopathic effects, such as cell shrinkage, membrane blebbing, and partial detachment, which became progressively evident with time and were more prominent after 48 and 72 h. These changes were comparable to those observed in the free DOX-treated group, where extensive cell rounding and loss of adherence were evident, consistent with strong cytotoxicity. Collectively, these morphological findings support the MTT results, demonstrating that the OA@ZnFe_2_O_4_ NPs are largely biocompatible while DOX loading significantly enhances their cytotoxic impact through sustained intracellular drug delivery.

As a next step, to investigate the impact of various treatments on the clonogenic capacity of SH-SY5Y neuroblastoma cells, a CFA was performed. As shown in both the representative CFA images and the quantitative bar chart (Figure 14), distinct differences were observed across the treatment groups.

**Figure 14:**
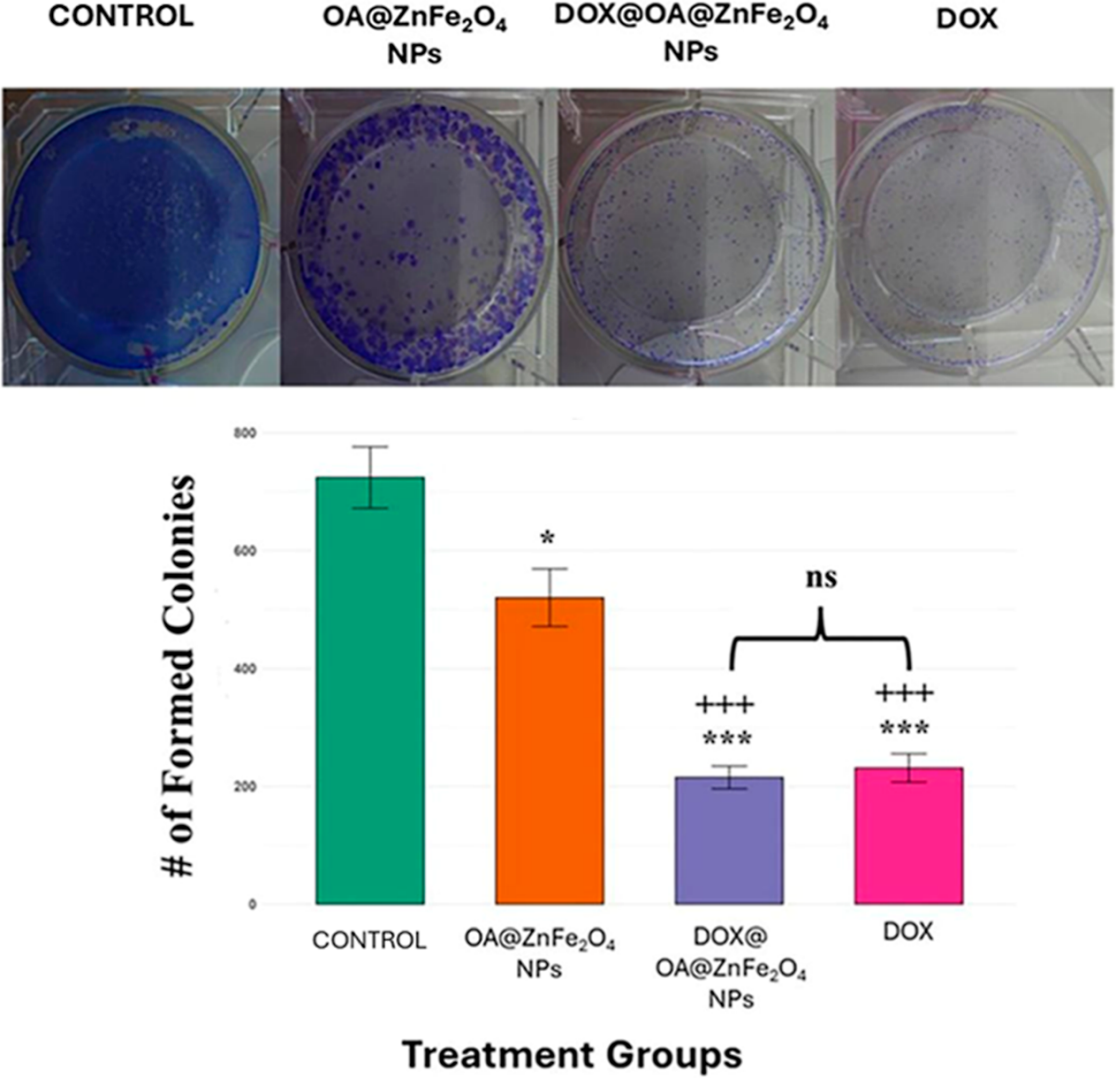
CFA of SH-SY5Y neuroblastoma cells treated with control (untreated), OA@ZnFe_2_O_4_ NPs, DOX@OA@ZnFe_2_O_4_ NPs, and soluble DOX. Top panel: representative images of stained colonies after 10 days of treatment. Bottom panel: quantification of colony numbers per well. Data are presented as mean ± SD from three independent experiments. Statistical comparisons with control are indicated by asterisks (*p < 0.05 and ***p < 0.001), while comparisons of DOX@OA@ZnFe_2_O_4_ NPs and DOX with NPs are denoted by plus signs (+++*p* < 0.001).

Untreated control cells formed 724 ± 52 colonies on average, whereas cells treated with the OA@ZnFe_2_O_4_ NPs alone showed a modest but statistically significant reduction in colony number (520 ± 49, *p* < 0.05 vs control), indicating a mild cytotoxic effect. Treatment with the DOX@OA@ZnFe_2_O_4_ NPs resulted in a profound inhibition of colony formation (215 ± 19, *p* < 0.001 vs control and vs NPs), closely mirrored by the group treated with soluble DOX (231 ± 24, *p* < 0.001 vs control and vs NPs). Notably, the difference between the DOX@OA@ZnFe_2_O_4_ NPs and soluble DOX was not statistically significant (*p* > 0.05), suggesting comparable levels of cytotoxic efficacy between the two DOX delivery methods.

The CFA highlights the differential impact of NP-based and conventional drug delivery systems on neuroblastoma cell survival. The slight but statistically significant reduction in colony number observed in the NP-only group underscores a degree of intrinsic cytotoxicity associated with the OA@ZnFe_2_O_4_ NPs, which is consistent with prior reports on the mild oxidative or metabolic stress induced by iron oxide-based nanomaterials.^72^ More strikingly, both DOX@OA@ZnFe_2_O_4_ NPs and soluble DOX led to robust inhibition of clonogenic potential, with reductions of over 65% relative to control. The comparable efficacy of the DOX@OA@ZnFe_2_O_4_ NPs and soluble DOX (*p* > 0.05) suggests that NP encapsulation does not compromise the cytotoxic activity of DOX in this context. However, the fact that the DOX@OA@ZnFe_2_O_4_ NPs performed at least as well as free DOX, combined with their potential for targeted delivery, reduced systemic toxicity, and improved pharmacokinetics, supports their further investigation as a promising vehicle for neuroblastoma therapy. Additionally, the significantly greater efficacy of the DOX@OA@ZnFe_2_O_4_ NPs compared to the OA@ZnFe_2_O_4_ NPs alone confirms that the observed cytotoxicity is predominantly attributable to DOX and not the nanocarrier itself.

ZnFe_2_O_4_ NPs possess superparamagnetic and pH-responsive properties that facilitate intracellular drug release once internalized into endosomal and lysosomal compartments, where the acidic environment (pH ≈ 5–5.5) promotes partial dissolution of the ferrite lattice and detachment of surface-bound DOX molecules.^53,73,74^ Consequently, released DOX diffuses to the nucleus and exerts its cytotoxic effect through DNA intercalation. This mechanism aligns with prior studies showing that ZnFe_2_O_4_-based nanocarriers enable pH-triggered intracellular release and enhanced nuclear accumulation of DOX in various cancer cell lines. ^73^ Thus, the obtained data from fluorescence imaging, MTT, microscopy, and CFA provide evidence for the internalization of the DOX@OA@ZnFe_2_O_4_ NPs and explaining the similarity in cytotoxic response between the DOX@OA@ZnFe_2_O_4_ NPs and free DOX treatments. The observed selectivity underscores the potential of the DOX@OA@ZnFe_2_O_4_ NPs as a safer and more efficient alternative to free DOX in neuroblastoma treatment.

## 4 Conclusion

In this study, OA@ZnFe_2_O_4_ NPs were successfully synthesized with a uniform cubic morphology, a single-phase spinel structure, and superparamagnetic behavior suitable for biomedical use. The OA@ZnFe_2_O_4_ NPs demonstrated an exceptional DOX loading efficiency of approximately 99%. They also exhibited a clear pH-responsive release behavior. Cellular imaging confirmed the efficient internalization and localization of the DOX@OA@ZnFe_2_O_4_ NPs in SH-SY5Y neuroblastoma cells. Biological assays revealed high biocompatibility of the OA@ZnFe_2_O_4_ NPs toward normal 3T3 fibroblasts and strong cytotoxic effects of DOX-loaded nanoparticles, comparable to free DOX. The cytotoxicity originated mainly from DOX release rather than the nanocarrier itself. Overall, the OA@ZnFe_2_O_4_ NPs represent a promising pH-sensitive and magnetically responsive platform for targeted neuroblastoma therapy, offering a high efficacy with reduced systemic toxicity.

## Supplementary Material



## Notes

Characterization data, supplementary figures, and calibration curves; SEM images of the OA@ZnFe_2_O_4_ and DOX@OA@ZnFe_2_O_4_ NPs; hydrodynamic size distributions obtained from DLS measurements under acidic and physiological conditions; and DOX calibration curves in PBS and citrate buffers (PDF)
