# Supplementary material for: Oleic Acid-Coated Zinc Ferrite Nanocubes: A Promising Nanocarrier for Neuroblastoma Therapy
Source: ACS Omega. 2025 Dec 15;11(6):9160–75. doi: 10.1021/acsomega.5c08155 (PMC12917698; doi:10.1021/acsomega.5c08155)
Supplement: Supplementary file 1 [file ao5c08155_si_001.pdf]

Supporting Information

Oleic Acid Coated Zinc Ferrite Nanocubes: A Promising Nanocarrier for Neuroblastoma Theraphy

Çiğdem Elif Akgün [a, b, *], *Fazilet Mısra Özdemir* [c], *İrem Abaka* [c], *Aydan Gülsu* [c], *Turan Demircan* [d, *]

a Research Laboratories Center, Muğla Sıtkı Koçman University, 48100, Menteşe, Muğla, Türkiye

b Department of Physics, Muğla Sıtkı Koçman University, 48100, Menteşe, Muğla, Türkiye

c Department of Molecular Biology and Genetics, Muğla Sıtkı Koçman University, 48100, Menteşe, Muğla, Türkiye

d Department of Medical Biology, Muğla Sıtkı Koçman University, 48100, Menteşe, Muğla, Türkiye

*Corresponding authors: Çiğdem Elif Akgün (elifdemirci@mu.edu.tr), Turan Demircan (turandemircan@mu.edu.tr)

1. Morphology of the DOX@OA@$ZnFe_2O_4$ nanoparticles

The surface morphology of the OA@$ZnFe_2O_4$ and DOX@OA@$ZnFe_2O_4$ NPs was examined to evaluate possible structural or aggregation changes after DOX loading. SEM images of the OA@$ZnFe_2O_4$ and DOX@OA@$ZnFe_2O_4$ NPs taken at different magnifications are presented in Figure S1 (a)-(d).

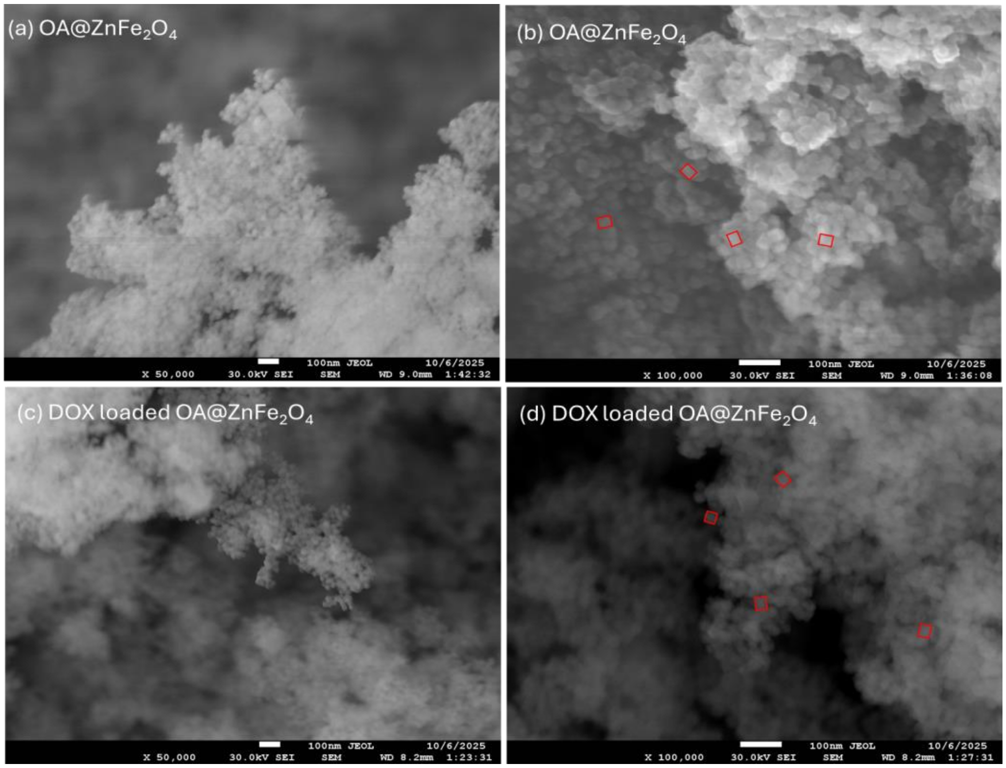

Figure S1. SEM images of the (a), (b) OA@$ZnFe_2O_4$ and (c), (d) DOX@OA@$ZnFe_2O_4$ NPs taken at different magnifications of x50,000 and 100,000.

As seen in Figure S1 (a)-(d), the OA@$ZnFe_2O_4$ and DOX@OA@$ZnFe_2O_4$ NPs revealed the well-defined cubic morphology of the nanoparticles with uniform particle size distribution. No significant morphological change was observed after DOX loading onto nanoparticles. This result confirms that DOX loaded without affecting the structure and morphology of the OA@$ZnFe_2O_4$NPs.

2. The dynamic light scattering (DLS) measurements

The hydrodynamic size distributions of the OA@$ZnFe_2O_4$ and DOX@OA@$ZnFe_2O_4$ NPs in PBS (pH 4.5) obtained to simulate acidic conditions is given in Figure S2 (a) and (b), respectively.

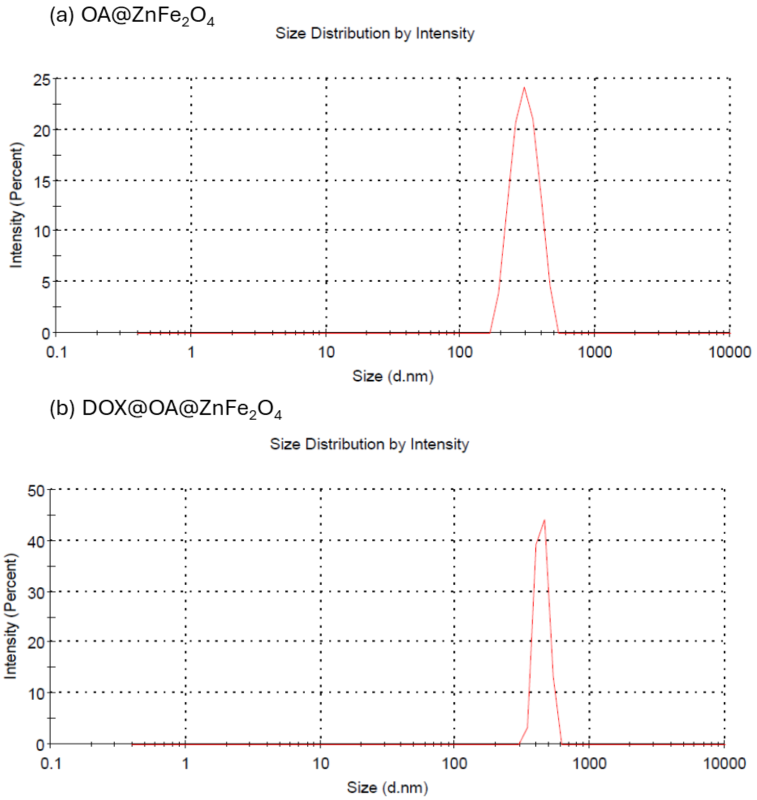

Figure S2. DLS size distribution of the (a) OA@$ZnFe_2O_4$ and (b) DOX@OA@$ZnFe_2O_4$ NPs in PBS (pH 4.5). The average diameters ($< D >$) with standard deviations (σ) and polydispersity index were found to be $< D > = 304.2 \pm 66.54$ nm, PDI=0.429 and $< D > = 439.9 \pm 48.38$ nm, PDI=0.562 for the OA@$ZnFe_2O_4$ and, DOX@OA@$ZnFe_2O_4$ NPs, respectively. The data corresponds to intensity-weighted distribution.

The hydrodynamic size distributions of the OA@$ZnFe_2O_4$ and DOX@OA@$ZnFe_2O_4$ NPs in DMEM (supplemented with 10% fetal bovine serum and %1 Penisilin/Streptomisin) obtained to simulate physiological conditions is given in Figure S3 (a) and (b), respectively. The data corresponds to number-, volume- and intensity-weighted distributions.

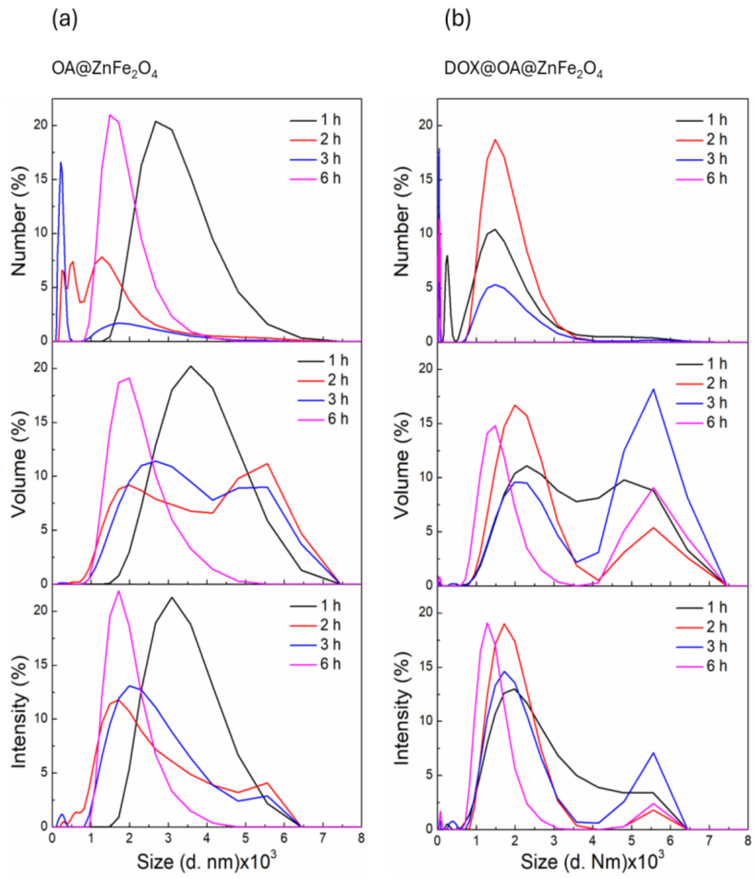

Figure S3. Hydrodynamic size distributions of the (a) OA@$ZnFe_2O_4$ and, (b) DOX@OA@$ZnFe_2O_4$ NPs in DMEM medium over incubation periods ranging from 1 to 6 h, obtained from DLS measurements. The data corresponds to number-, volume- and intensity-weighted distributions.

3. DOX calibration curve

The standard calibration curves were prepared using a series of DOX solutions with known concentratioins in PBS and citrate buffers, as shown in Figure S4 (a)-(b).

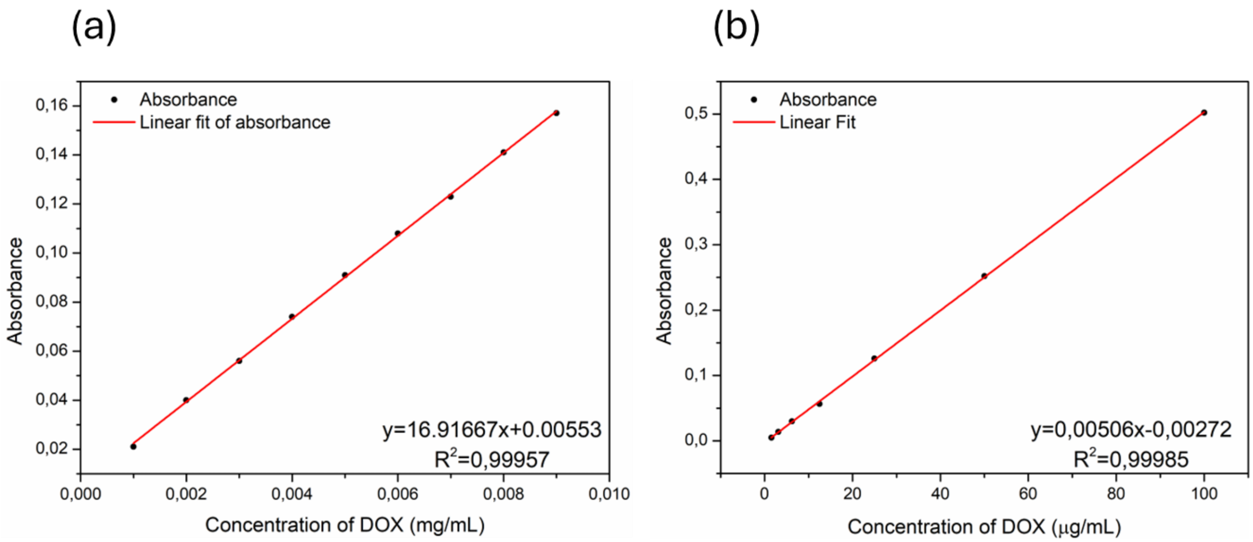

Figure S4. DOX calibration curves in (a) PBS, and (b) citrate buffer.
